# Identifying human diamine sensors for death related putrescine and cadaverine molecules

**DOI:** 10.1371/journal.pcbi.1005945

**Published:** 2018-01-11

**Authors:** Cristina Izquierdo, José C. Gómez-Tamayo, Jean-Christophe Nebel, Leonardo Pardo, Angel Gonzalez

**Affiliations:** 1 Laboratori de Medicina Computacional, Unitat de Bioestadística, Facultat de Medicina, Universitat Autònoma de Barcelona, E-08193 Bellaterra, Spain; 2 Faculty of Science, Engineering and Computing, Kingston University, London, United Kingdom; Icahn School of Medicine at Mount Sinai, UNITED STATES

## Abstract

Pungent chemical compounds originating from decaying tissue are strong drivers of animal behavior. Two of the best-characterized death smell components are putrescine (PUT) and cadaverine (CAD), foul-smelling molecules produced by decarboxylation of amino acids during decomposition. These volatile polyamines act as ‘necromones’, triggering avoidance or attractive responses, which are fundamental for the survival of a wide range of species. The few studies that have attempted to identify the cognate receptors for these molecules have suggested the involvement of the seven-helix trace amine-associated receptors (TAARs), localized in the olfactory epithelium. However, very little is known about the precise chemosensory receptors that sense these compounds in the majority of organisms and the molecular basis of their interactions. In this work, we have used computational strategies to characterize the binding between PUT and CAD with the TAAR6 and TAAR8 human receptors. Sequence analysis, homology modeling, docking and molecular dynamics studies suggest a tandem of negatively charged aspartates in the binding pocket of these receptors which are likely to be involved in the recognition of these small biogenic diamines.

## Author summary

The distinctive dead smell comes largely from molecules like cadaverine and putrescine that are produced during decomposition of organic tissues. These volatile compounds act as powerful chemical signals important for the survival of a wide range of species. Previous studies have identified the trace amine-associated receptor 13c (or TAAR13c) in zebrafish as the cognate receptor of cadaverine in bony fishes. In this work, we employed computational strategies to disclose the human TAAR6 and TAAR8 receptors as sensors of the putrescine and cadaverine molecules. Our results indicate that several negatively charged residues in the ligand binding pocket of these receptors constitute the molecular basis for recognition of these necromones in humans.

## Introduction

Olfaction is the major neurosensory function by which many species explore the chemical composition of their natural environments to locate food, avoid potentially harmful situations, recognize territory, identify members of their own group or predators, and choose a mate. Notable among the many olfactory signals is the characteristic pungent odor of a decaying cadaver. The smell of death consists of a complex mixture of volatile organic compounds [1]. Two of the most significant components of the ‘rotting flesh’ odor are putrescine (PUT) and cadaverine (CAD), early described in 1885 by the German physician Ludwig Brieger [2]. PUT and CAD are diamine products of decarboxylation of the amino acids lysine and arginine during decomposition of animal tissue. Both have short hydrocarbon chains with a primary amine group at each end. PUT has four carbon atoms (C4) in the chain between the two amines, whereas there are five carbon atoms (C5) in CAD. These molecules, characterized by a foul-smelling odor that repels most animals, could also act as an attractant for scavengers, parasites and others [3–5].

Recent studies in mouse and fish indicate that CAD activates chemosensory receptors in the olfactory epithelium, called trace amine-associated receptors (TAARs) [6–8]. TAAR genes are found in all vertebrate taxa, varying in number between species, and constitute a sensory subsystem to detect volatile molecules complementary to the canonical olfactory receptors (ORs) [9] and pheromone vomeronasal receptors (VRs) [10]. These membrane proteins generally recognize volatile amines linked to stress, social cues and predator-derived chemicals [11–13]. TAARs belong to family A of G-protein-coupled receptors (GPCR), which are characterized by the transduction of sensory signals of external origin through second messenger cascades controlled by different heterotrimeric guanine nucleotide binding proteins (G-proteins) coupled at their intracellular regions [14]. The predominant signaling pathway described for these receptors involved the Gα_olf_ activation, increasing cAMP levels upon stimulation by trace amines [9, 15]. Thus, TAAR responses are likely mediated by coupling to the canonical odorant transduction cascade, acting on cyclic nucleotide-gated ion channels which allow Na^+^ and Ca^2+^ ions to enter into the cell, depolarizing olfactory sensory neurons (OSNs) and beginning an action potential which carries the information to the brain [16].

TAARs share a strong evolutionary relationship with biogenic amine GPCRs such as the serotoninergic (5-HTR), β-adrenergic (ADRB) dopaminergic (DRD) and histaminergic (HRH) receptors [17]. These receptors are characterized by a highly conserved molecular architecture of seven α-helical transmembrane (7-TM) segments connected to each other by three extracellular loops (3-ECL) and three intracellular loops (3-ICL) [18]. X-ray 3D structures of several aminergic GPCRs have revealed topological conserved positions in the TM helix bundle that are critical for ligand-receptor interactions [19]. Particularly, a conserved aspartic acid at position 3.32 in TM3 [number correspond to Ballesteros-Weinstein nomenclature [20]] forms a salt bridge with the positively charged nitrogen of aminergic compounds, and polar residues at positions 5.42, 5.43 and/or 5.46 in TM5 form hydrogen bond interactions with weakly acidic hydroxyl moieties of several ligands. An interesting example in this respect is the presence of two aspartates (Asp_3.32_ and Asp_5.42_) essential for the binding of histamine and other dicationic at low pH ligands to the non-chemosensory histamine receptor type-2 (HRH2) [21, 22].

Most mammalian TAARs, and some from teleosts retain the negatively charged Asp_3.32_, which supports its role for volatile amine recognition [12]. Among these, a small group of TAARs contain a second aspartate at position 5.42 or 5.43 (zebrafish: zTAAR13c, zTAAR13d; human: hTAAR6, hTAAR8; mice: mTAAR6, mTAAR8b; and others). One of the few studies that explored the impact of these two negative charges in the binding of ligands it was shown that CAD binds zTAAR13c via two ionic interactions between the protonated amine and Asp_3.32_ and Asp_5.42_ [23]. However, despite the theoretical and empirical importance of this finding, very little is reported in the literature for how PUT or CAD exert their effects, and the TAAR family remain largely understudied compared to other GPCR subfamilies. Following the working hypothesis of the involvement of TAARs in death-odor detection, we have investigated the molecular interactions of PUT and CAD with the hTAAR6 and hTAAR8. The results of molecular modeling and docking experiments, in addition to unrestrained microsecond-scale (μs) molecular dynamics (MD) simulations indicate that PUT and CAD fit into the binding pocket of the human TAAR6 and TAAR8, making stable interactions with Asp_3.32_ and Asp_5.43_. This finding supports the importance of the conserved tandem of negatively charged residues in the orthosteric cavity of these receptors, offering a robust modelling hypothesis for the recognition of C4 and C5 diaminated compounds. A structure-informed multiple sequence alignment of several TAARs from well-known classes of vertebrates reveals the conservation of both aspartates in at least one of either TAAR6 or TAAR8 homolog of most mammals, while being absent in amphibians, reptiles and birds.

## Results

### A tandem of conserved aspartates in the binding pocket of bony fishes TAAR13c and mammalian TAAR6 and TAAR8

**Fig 1:**
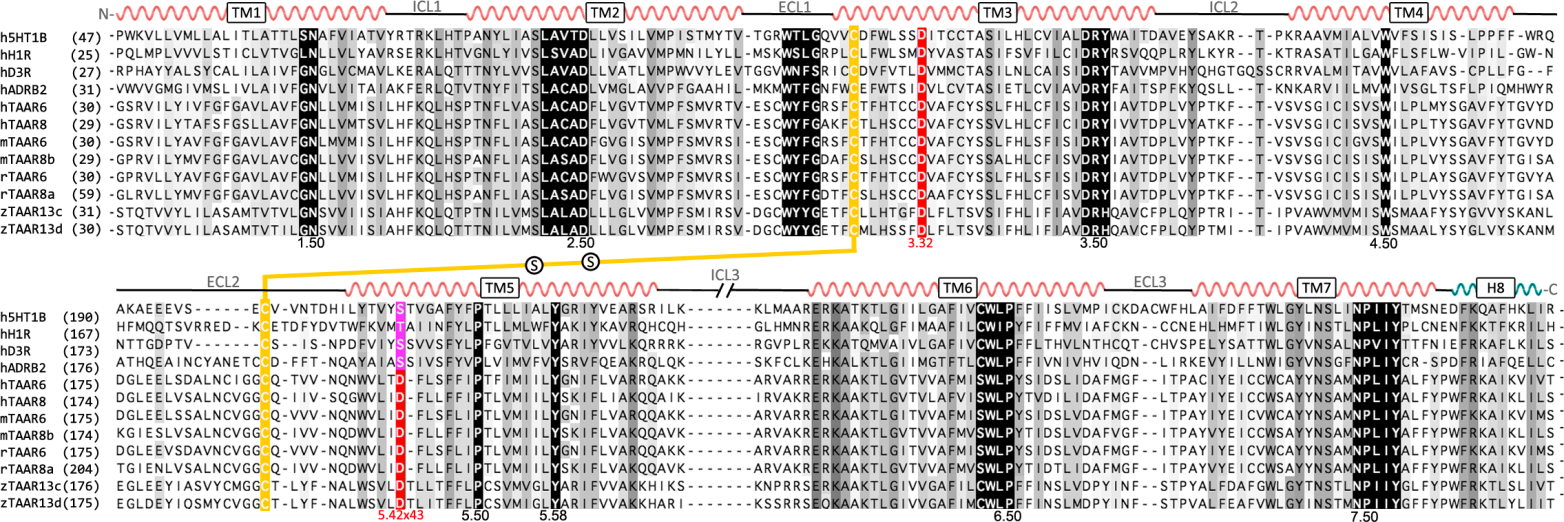
Multiple sequence alignment of representative aminergic receptors and selected TAARs from different organisms. Positions that are at least 30% or 95% conserved are highlighted in gray and black, respectively. Highly conserved residues in the class A GPCR family are indicated by the Ballesteros-Weinstein numbering (X.50 of helix X), as well as the conserved cysteines involved in disulfide bridges (in yellow). Residues at position 3.32 and 5.42/5.43 are highlighted in red. Non-conserved N-, C- terminal and ICL3 amino acid sequences are omitted from the figure. *Acronyms*: h5HT1B (human 5-hydroxytryptamine receptor 1B), hH1R (human histamine receptor H1), hD3R (human dopamine receptor D3), hADRB2 (human β_2_-adrenergic receptor), hTAAR6 (human trace-amine associated receptor 6), hTAAR8 (human trace-amine associated receptor 8), mTAAR6 (mouse trace-amine associated receptor 6), mTAAR8b (mouse trace-amine associated receptor 8b), rTAAR6 (rat trace-amine associated receptor 6), rTAAR8a (rat trace-amine associated receptor 8a), zTAAR13c (zebrafish trace-amine associated receptor 13c) and zTAAR13d (zebrafish trace-amine associated receptor 13d).

Numerous structural studies of GPCRs have revealed a strong conservation of the 7-TM helical architecture, as well as in a number of topologically equivalent residues involved in the binding of ligands [24]. This information has been integrated in Multiple Sequence Alignments (MSAs) in order to identify functional amino acids, localize amino acid insertions and deletions or improve classification [25–27]. Fig 1 shows a structure-based MSA of representative biogenic amine receptors, including the structurally determined 5-HT1BR (PDB ID: 4IAR), ADRB2 (2RH1, 3P0G), D3R (3PBL), H1R (3RZE) and selected TAAR6, TAAR8, TAAR13c and TAAR13d sequences from different organisms (see S1 Fig for an extensive list). The sequence similarity between members of the distinct subfamilies (*e*.*g*. TAARs *vs*. 5-HTRs *vs*. ADRBs *vs*. DRDs *vs*. HRHs) is ∼30%, which is archetypal of class A GPCRs despite their high structural resemblance [28]. Nonetheless, all sequences display well-known consensus signatures GN_1.50_, LAxxD_2.50_, DR_3.50_Y, W_4.50_, P_5.50_, Y_5.58_, CWxP_6.50_, NP_7.50_xxY [18], including the ECL1 WxFG motif and the highly conserved cysteines in TM3 and ECL2 involved in a disulfide bridge for the majority of class A GPCRs [29].

The key Asp_3.32_, directly involved in the interaction with aminergic ligands, aligns in all sequences. In addition, a second aspartate (Asp_5.42_ or Asp_5.43_, according to the receptor type) is present on TAAR13c, TAAR13d, TAAR6 and TAAR8 sequences (Fig 1 and S1 Fig). Both positions are an integral part of the orthosteric-binding site in most aminergic receptors and are frequently involved in interactions with polar groups of substrates [19]. In the MSA of Fig 1, the Asp_5.42_ of the teleost fish TAAR13c and TAAR13d sequences is aligned with Asp_5.43_ of mammalian TAAR6 and TAAR8 by the introduction of a single gap in the MSA. The occurrence of such a gap has been described before in order to amend non-matching amino acids due to local distortions in the α-helical scaffold [25]. In this particular case, we considered that the negatively charged aspartate in TM5 might be similarly positioned to recognize chemicals of comparable size and with two positively charged groups.

### The orthosteric site of human TAAR6 and TAAR8 and location of conserved aspartates

Currently, there is no experimental structural data of any TAAR in complex with their cognate substrate. However, the recent breakthroughs in GPCR structure determination [30] allow us to study the molecular basis of their interactions using modeling with high quality, structurally close, templates. Here, we used a structure-based MSA (Fig 1), together with the experimentally determined three-dimensional (3D) atomic coordinates of the ADRB2 in active and inactive conformational states [31, 32], to construct molecular 3D-models of human TAAR6 and TAAR8. From a total of 400 generated models, four representative structures of the agonist bound active- (hTAAR6_active-like_/hTAAR8_active-like_) and inactive- (hTAAR6_inactive-like_/hTAAR8_inactive-like_) conformations were selected based on their stereochemical quality and subsequently refined by molecular dynamics simulations (S1 Table). In addition, for comparison purposes, computational models of zebrafish TAAR13c were developed using the same methodology (see Methods).

**Fig 2:**
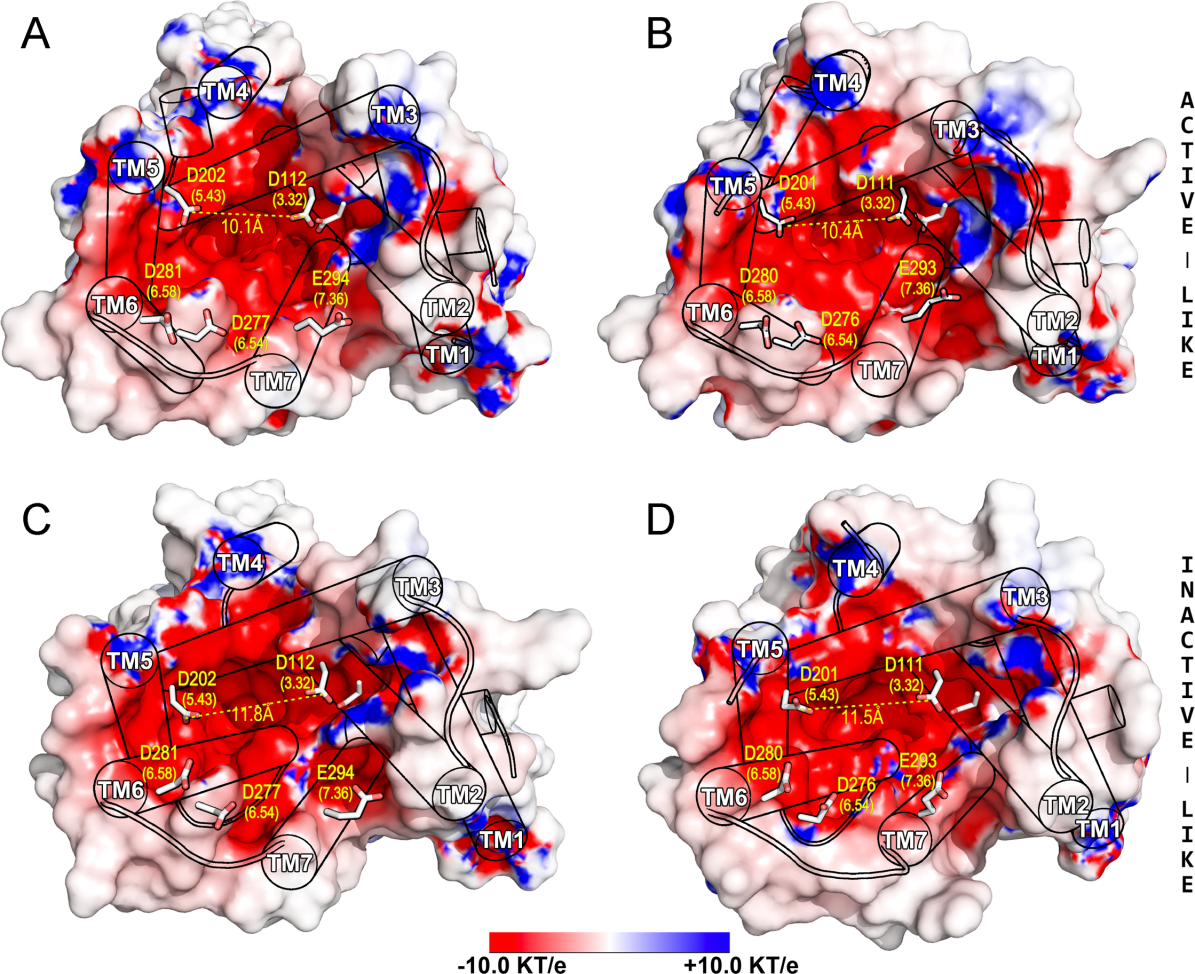
The orthosteric ligand-binding pocket of human TAAR6 and TAAR8. Surface representation of the molecular models of hTAAR6 (A and C) and hTAAR8 (B and D) in the active- (top panels) and inactive-like (bottom panels) conformations. Extracellular view of the identified ligand binding cavities with molecular surfaces colored by the electrostatic potential calculated using the program APBS with nonlinear Poisson-Boltzmann equation and contoured at ±10 kT/e (negatively and positively charged surface areas in red and blue, respectively). Residues contributing to the electronegative potential of the binding pocket are represented in sticks and numbered according to the receptor type (Ballesteros-Weinstein scheme in parenthesis). Calculated distances between carboxyl moieties of Asp_3.32_ and Asp_5.43_ are shown for each molecular structure (yellow dashed lines). Protein backbones are shown in cylinders except ECL2 conformations (here omitted for clarity).

To a great extent, active- and inactive-like human TAARs models displayed a high similarity in the extracellular ligand-binding region (average root mean square deviation RMSD < 2.0 Å), whereas major differences were located at the cytoplasmic G protein-coupling domain. In this region, outward displacements of the TM5 (∼5.0 Å) and TM6 (∼10.0 Å) necessary for coupling the G-protein-mimetic nanobody differentiate the TAAR6_active-like_/TAAR8_active-like_ from the TAAR6_inactive-like_/TAAR8_inactive-like_ structures (S2 Fig). Analysis of the biogenic amine GPCRs topologically equivalent ligand-binding pocket (region comprising TMs 3–7) in the hTAAR6, hTAAR8 and zTAAR13c molecular models clearly shows a strong electronegative character (Fig 2 and S3 Fig). An exceptional cluster of six conserved Asp/Glu residues on the TMs contributed to the overall negative electrostatic potential of the binding cavity (Asp_3.32_, Asp_5.43_, Asp_6.54_, Asp_6.58_ and Glu_7.36,_ identified in Fig 2 and S1 Fig). It has been shown that the presence of charged residues at the orthosteric binding site entrance of GPCRs serve as a floodgate to remove the water solvent shell around ligands during the process of transferring from the extracellular aqueous environment to the binding site crevice in the TM domain [33–35]. This is of particular relevance for dicationic ligands as PUT and CAD. Thus, we hypothesized that the amino acids at the extracellular entrance playing this role are Asp_6.54_ (hTAAR6 D277; hTAAR8 D276), Asp_6.58_ (hTAAR6 D281; hTAAR8 D280) or/and Glu_7.36_ (hTAAR6 E293; hTAAR8 E294). On the other side, we assumed that Asp_3.32_ (hTAAR6 D112; hTAAR8 D111) and Asp_5.43_ (hTAAR6 D202; hTAAR8 D201) located at the same height at the bottom of the TM helix cavity, serve as the final anchor points of PUT and CAD (see below).

### Computational study of molecular interactions of PUT and CAD with human TAAR6 and TAAR8

**Fig 3:**
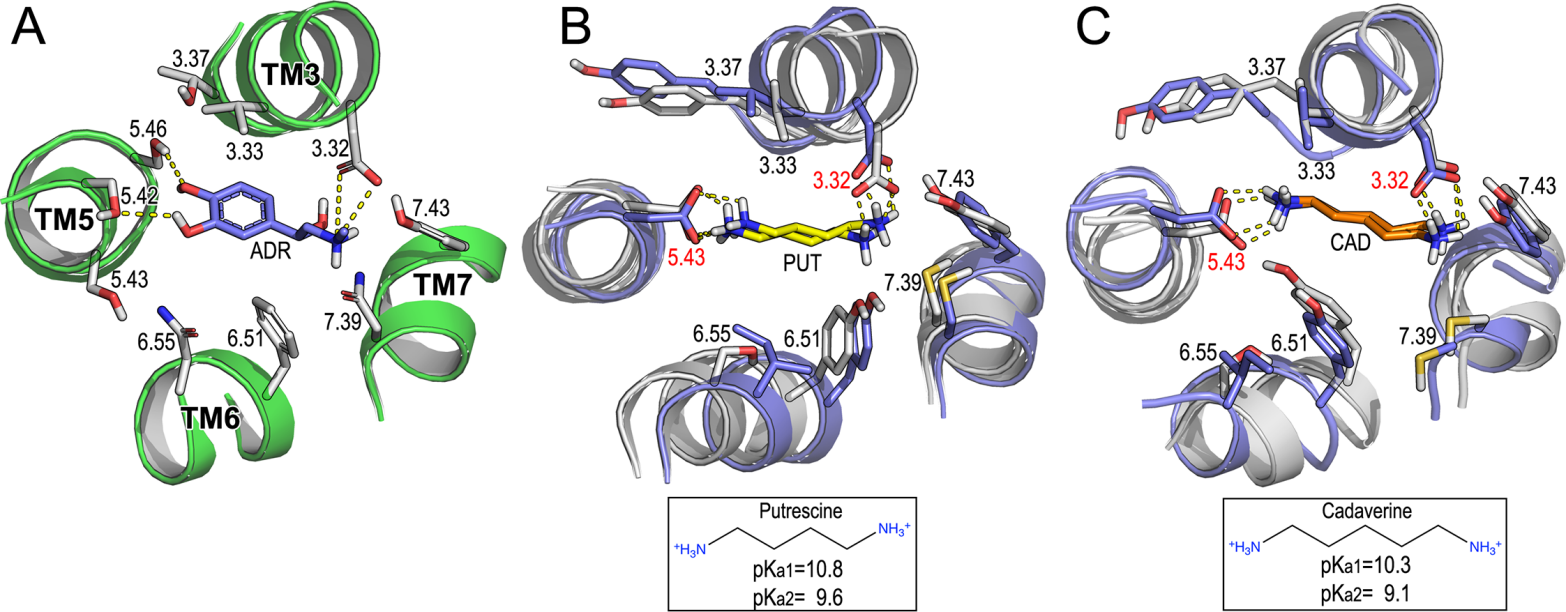
Molecular interactions of PUT and CAD with human TAAR6 and TAAR8. (A) Key features of the full agonist adrenaline (ADR, blue sticks) in the binding pocket of the ADRB2 active structure (PDB ID:4LDO; region comprising the TMs 3-5-6-7 in green ribbons). (B) Superposition of molecular docking of putrescine (PUT, yellow sticks), and (C) cadaverine (CAD, orange sticks), in the active-like TAAR6 (light-gray ribbons) and TAAR8 (light-blue ribbons) molecular models. Contact residues at a distance < 3.5 Å from ligands are shown in sticks and numbered according to Ballesteros-Weinstein numbering. Predicted (ligand–receptor) hydrogen bonds and salt bridge interactions are shown in dashed lines. The 2D chemical structure of PUT and CAD protonated at physiological pH, with estimated pKa1 and pKa2 for each amino group are indicated at the bottom of panels B and C, respectively.

PUT and CAD are chemically very similar: they are symmetrical molecules with short hydrocarbon chains (C4 & C5 carbon atoms, respectively) and two primary amine groups at each end (average length between nitrogen atoms is 6.3 and 7.4 Å, respectively) (Fig 3). These compounds are smaller than classical aminergic ligands. Thus, owing to the fact that zebrafish TAAR13c has been identified as a high-affinity receptor for the odd-chained diamines CAD (C5) and diaminoheptane (C7) [23], it is reasonable to assume that the shorter PUT and CAD could also fit in the binding pocket of human TAAR6 and TAAR8. To test this hypothesis, we conducted molecular docking experiments of PUT and CAD to the hTAAR6 and hTAAR8 (Fig 3 and S4 Fig). As depicted in Fig 3, the chosen orientations of both molecules in the TAAR6 and TAAR8 was similar to that observed in the adrenaline-activated structure of ADRB2 [36]. The main interactions involved are a double salt-bridge between PUT/CAD protonated amines and carboxylic groups of Asp_3.32_/Asp_5.43_, and hydrophobic contacts with V_3.33_ (hTAAR6 V113; hTAAR8 V112) and Y_6.51_ (hTAAR6 Y274; hTAAR8 Y273) in close proximity to the central alkyl chains of the ligands. Likewise, similar molecular poses and score energies were obtained for the zTAAR13c bound to CAD (S2 Table and S5 Fig) that, as mentioned earlier, has been experimentally demonstrated.

Unbiased 1μs MD simulations of the ligand-receptor systems were conducted in an explicit lipid bilayer environment to assess the stability of the proposed binding: hTAAR6_active-like_/PUT; hTAAR6_active-like_/CAD; hTAAR6_inactive-like_/PUT; hTAAR6_inactive-like_/CAD; hTAAR8_active-like_/PUT; hTAAR8_active-like_/CAD; hTAAR8_inactive-like_/PUT, hTAAR8_inactive-like_/CAD and compared with the zTAAR13c_active-like_/CAD and zTAAR13c_inactive-like_/CAD binding complexes (S3 Table). For the active-like conformations, the MD systems included a receptor-specific nanobody Nb80 with G-protein-like properties [32], coupled to the intracellular part of the receptors (S2 and S6 Figs). This procedure is necessary as agonists are incapable of stabilizing the fully active conformation of the receptor in the absence of the G protein or a G-protein-mimetic nanobody [37, 38]. All MD simulations gave rise to stable trajectories and membrane-protein systems remained steady after relaxation and during the data collection steps. The root mean square deviation (RMSD_backbone_ < 4.0 Å) in all simulated systems demonstrates the overall structural stability of the modeled receptors. Likewise, the accuracy of the docking poses was confirmed by the small fluctuations of ligands coordinates, in particular for the active-like structures (S7 and S8 Figs). These results support the hypothesis that both natural diamines are likely to interact in a stable manner with human TAAR6 and TAAR8 in the same way as CAD to the zebrafish TAAR13c.

**Fig 4:**
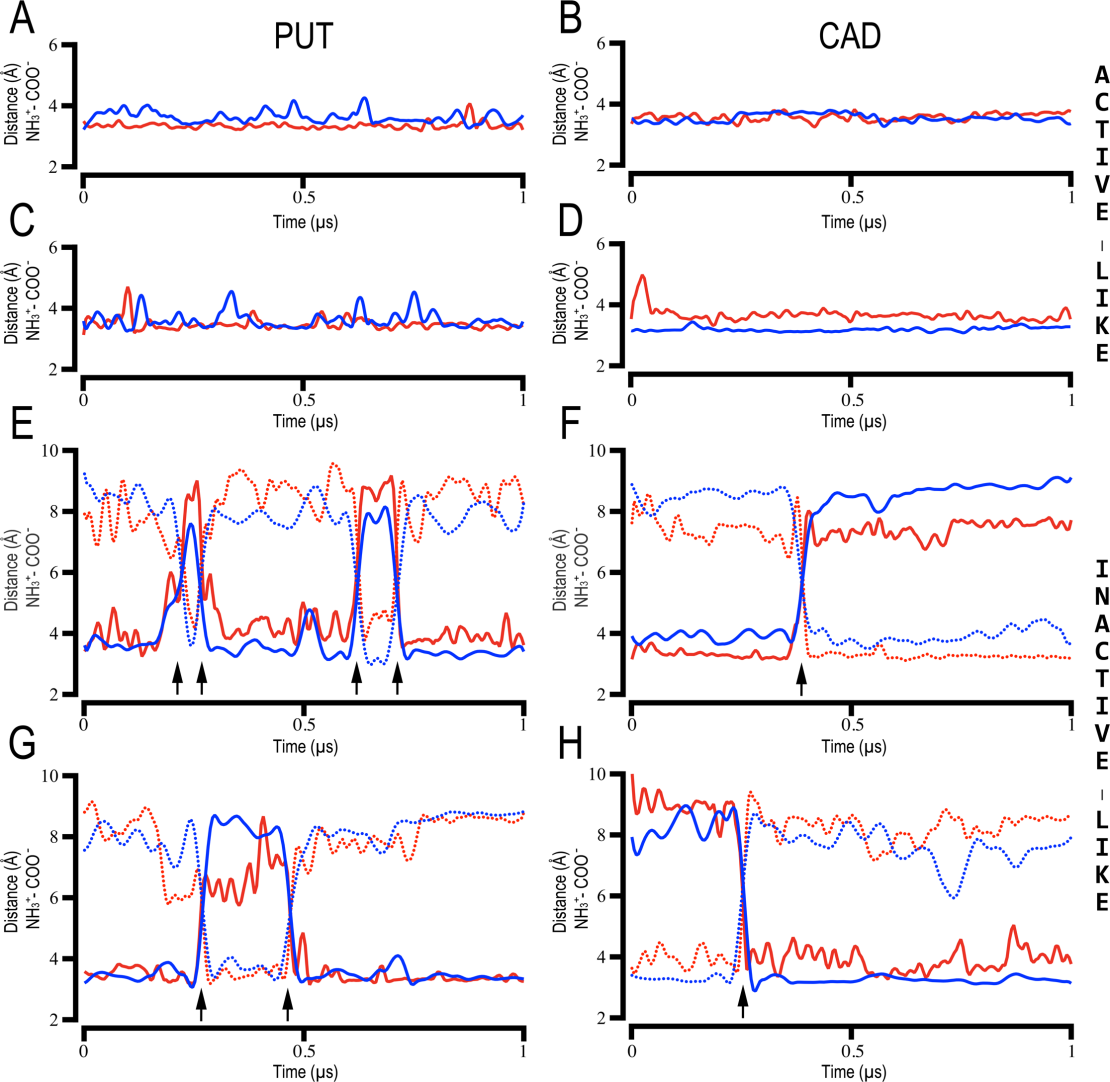
Stability of interactions between PUT and CAD and human TAAR6 and TAAR8. Time evolution (x-axis) of intermolecular distances (y-axis) between Asp_3.32/5.43_ (-COO^-^) and PUT/CAD (-NH3^+^) in 1.0 μs unbiased MD simulations. Each plot corresponds to one of the eight simulated ligand-receptor molecular complexes: hTAAR6_active-like_/PUT (A), hTAAR6_active-like_/CAD (B), hTAAR8_active-like_/PUT (C), hTAAR8_active-like_/CAD (D), hTAAR6_inactive-like_/PUT (E), hTAAR6_inactive-like_/CAD (F), hTAAR8_inactive-like_/PUT (G), hTAAR8_inactive-like_/CAD (H). Continuous and dotted lines correspond to distances between N1 and N2 atoms of the ligands with Asp_3.32_ (red) and Asp_5.43_ (blue) carboxyl groups, respectively. Black arrows at the bottom indicate the flip-transitions (180° rotation) of PUT and CAD in the binding pocket of the inactive-like models.

Fig 4 shows the computed distances between the nitrogen atom of the protonated amines of PUT/CAD and the carboxylate groups of Asp_3.32_/Asp_5.43_ in the human TAAR6 and TAAR8 along the MD trajectories. Clearly, in the inactive-like models these distances fluctuate through the simulations, revealing that PUT/CAD could spin around inside the binding pocket (Fig 4E–4H). These flip- transitions occur very rapidly (~10ns on average) and are quickly stabilized by salt-bridges with the opposite pairs of the interacting partners. Notably, this effect is not observed in the active-like models (Fig 4A–4D), probably due to the small contraction of the orthosteric cavity observed in the activated state of the receptors [39] that impedes the transition. This is reflected in the initial homology models, depicted in Fig 2, in which the distances between the carboxyl moieties of Asp_3.32_/Asp_5.43_ were ~1.0 Å smaller in the active-like conformations (average dist. 10.2 Å) with respect to the inactive ones (average dist. 11.6 Å). A similar trend was observed in the zTAAR13c/CAD complexes (S3 and S8 Figs). In all cases, the TM3-TM5 distance was further reduced during the MD trajectories, dropped below 10 Å in the active-like ligand-receptor simulated complexes (S3 Table).

**Fig 5:**
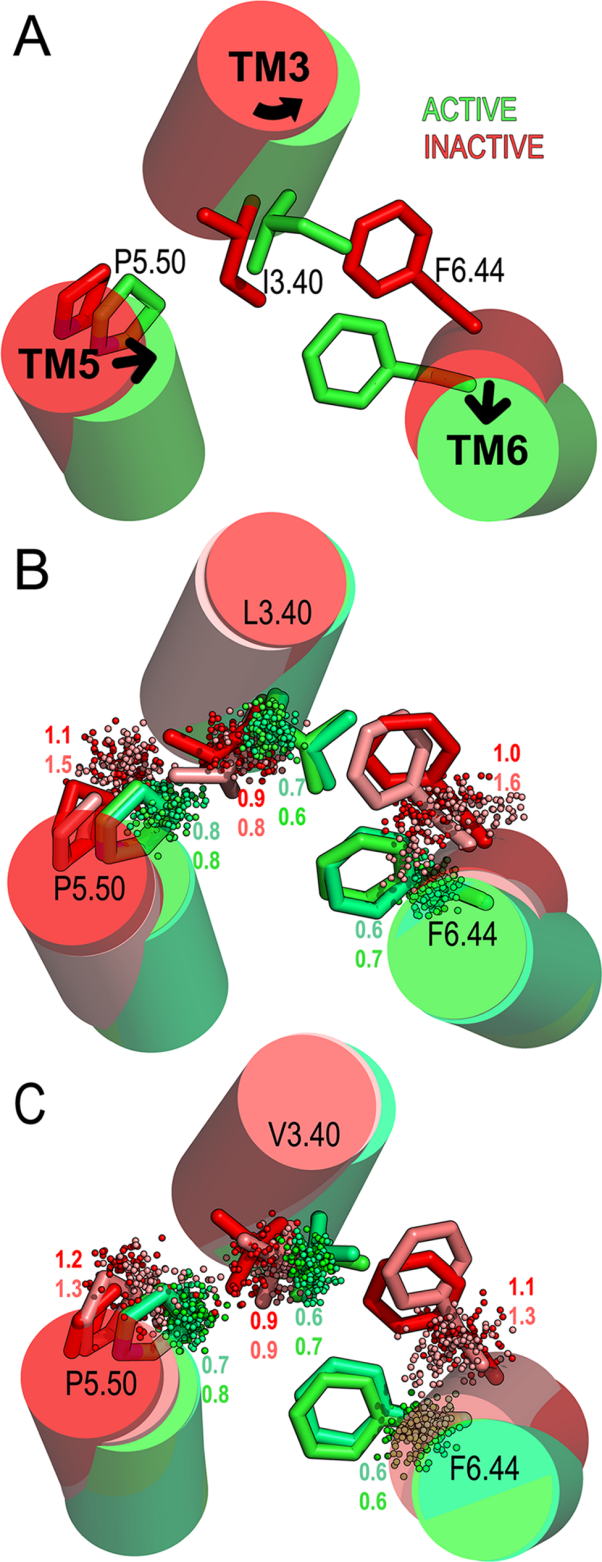
Effect of PUT and CAD on the ‘transmission switch’ amino acids. (A) Structural attributes of the ‘transmission switch’ residues 3.40, 5.50 and 6.44 (in sticks), in TMs 3-5-6 (in cylinders), on the ADRB2 in agonist bound active- (PDB ID: 3SN6, in green) and inverse agonist bound inactive- conformation (2RH1, in red). Arrows represent the observed movement of the helices in the transition from the inactive to the active state of the receptor. (B) Distribution of L_3.40_, P_5.50_ and F_6.44_ Cβ atoms positions (dots) in the TAAR6 and (C) V_3.40_, P_5.50_ and F_6.44_ Cβ atoms in the TAAR8 during simulations of active-like PUT/CAD bound in green/light green and inactive-like PUT/CAD bound in red/light red. Numbers correspond to the standard deviation (SD) of the Cβ atoms positions from the centroid of 100 evenly spaced snapshots extracted from the 1.0 μs of unbiased MD simulations.

Furthermore, we analyzed in the MD simulations of active- and inactive-like structures the ‘transmission switch’, comprising amino acids at positions 3.40, 5.50, and 6.44 (Fig 5 and S9 Fig). These residues located below the ligand binding cavity adopt different conformations upon binding of agonists, inverse agonists or allosteric modulators, and thus constitute a good model to study the effect of the ligands on the conformational states of the receptors [24, 38, 40, 41]. Similarly to the agonist-bound ADRB2 in complex with G_αs_ (Fig 5A in green), the TAAR6/TAAR8 active-like complexes (green in Fig 5B and 5C) were characterized by the inward displacement of TM5 at the highly conserved Pro_5.50_ (hTAAR6 P209; hTAAR8 P208), steric competition with bulky hydrophobic residues (hTAAR6 L120; hTAAR8 V119) at position 3.40 and small counterclockwise rotation of TM3 which leads to a steric exclusion with the side chain of F_6.44_ (hTAAR6 F267; hTAAR8 F266) and outward displacement of TM6. Conformational sampling analysis of these residues revealed higher fluctuations in the inactive-like complexes, in particular P_5.50_ and F_6.44_ (standard deviations (SD) of Cβ atoms position ≥ 1Å, Fig 5B and 5C in red/light red) with regard to the active-like complexes (SD of Cβ < 1Å, Fig 5B and 5C in green/light green). We believe this is a consequence of the disrupted interactions between PUT and CAD with Asp_3.32_ and Asp_5.43_ (Fig 4E–4H). This is in contrast to the strong binding in the active-like receptors (Fig 4A–4D), which suggest that both ligands contribute to the constriction of the binding cavity through stable ionic interactions with the Asp_3.32_/Asp_5.43_ pair, stabilizing active conformations same as agonists compounds [39] and consistent with previous observations in the zTAAR13c [7].

### Identifying TAAR6 and TAAR8 related orthologs as diamine sensors in mammals

**Fig 6:**
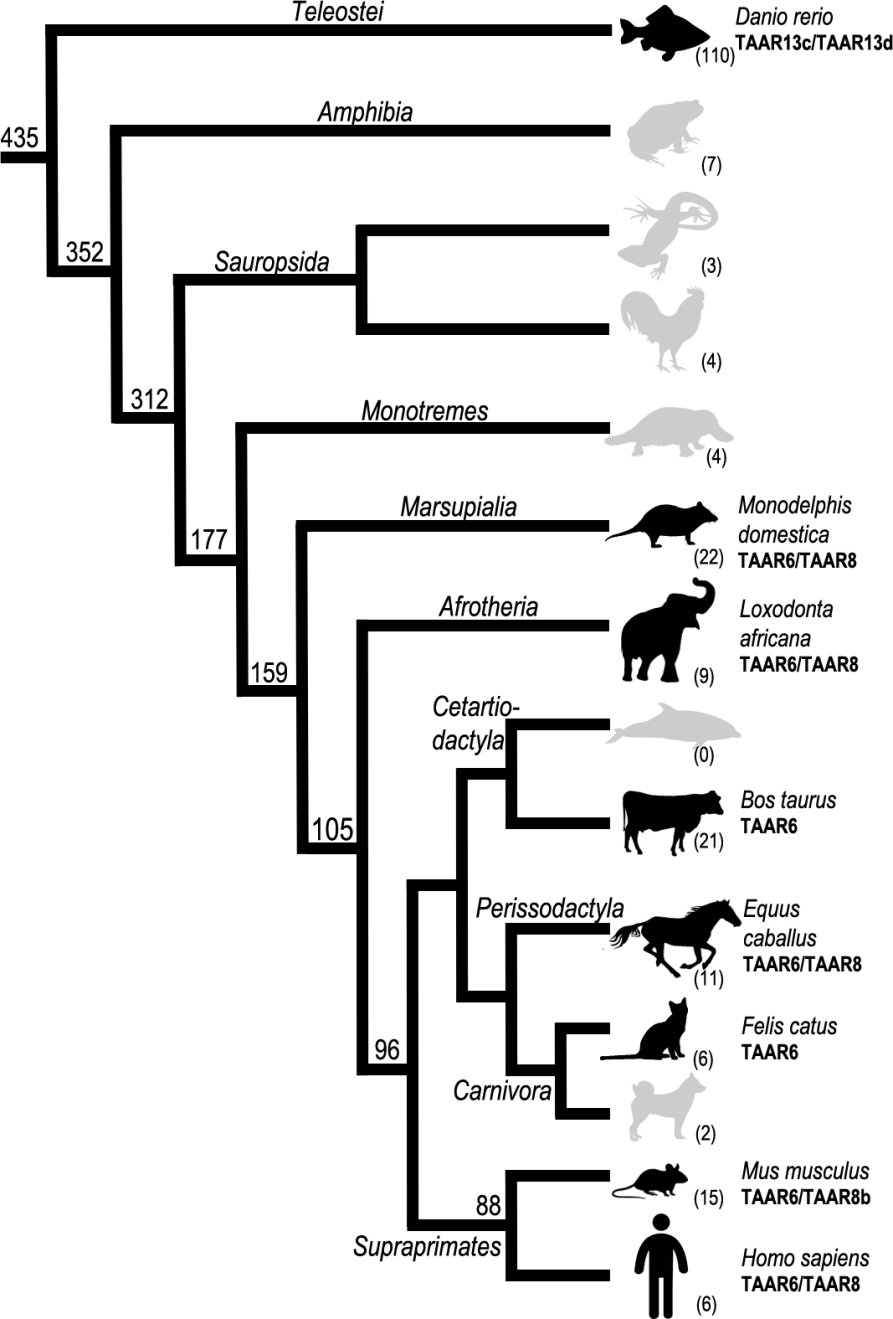
Cladogram representing the presence of TAARs in a consensus phylogeny of different vertebrates. The total number of functional TAAR genes is shown in parenthesis for each organism. Teleost TAAR13c/TAAR13d with proven affinity for CAD/PUT, respectively and therian-specific TAAR6/TAAR8 with the conserved tandem of aspartates in the TM3 and TM5 appear in bold (corresponding to black silhouettes in the species of origin). Approximate divergence times between species (million years ago; MYA) are shown in the internal nodes.

In addition to TAARs, the chemosensory function in vertebrates it is carried out by ORs, VRs and taste receptors (TRs) GPCR subfamilies. The number of genes and pseudogenes of these chemosensory receptors, as well as their associated sensory organs, vary enormously among species according their different living environments [42, 43]. Likewise, the TAAR gene repertoire is highly variable among vertebrate taxa [44]. Copy number of TAARs ranges over a hundred in teleosts (zebrafish), to less than ten in amphibians (clawed frog), and only a few (1 to 4) in sauropsids (zebra finch, anole lizard and chicken). The number of TAARs in synapsids is generally larger than in other four-limbed vertebrates, but also varies significantly across species, even within the same taxonomic group (see Fig 6). We searched for the tandem of aspartates in 220 identified vertebrate TAARs [44], and except for the teleosts TAAR13a, TAAR13c, TAAR13d, TAAR13e, TAAR14d and therian TAAR6 and TAAR8 sequences, no other receptor with two conserved negatively charged residues in the TM3 and TM5 helices was found in the monotreme, sauropsid or amphibian lineages.

It has been reported that the identified zTAARs could detect chemicals with two cations. In particular, CAD binds to the zTAAR13c with μM affinity [7], whereas PUT and CAD bind with different affinities to the zTAAR13d [23]. Similarly, mutation of either Asp_3.32_ or Asp_5.42_ in these receptors reduced or abolished responses to dicationic ligands. On the other hand, TAAR6 and TAAR8 homologous genes with conserved Asp_3.32_/Asp_5.43_ were found in most of placental mammals including terrestrial ungulates (hoofed animals), supraprimates (human, mouse, rat), carnivores (with a notable exception in dogs), and were absent in cetaceans (see Figs 6 and S1). Frequently, these two genes are contiguously located in chromosomal regions (16.6kb distance between hTAAR6 and hTAAR8 on human chromosome 6), which suggests they are products of genome duplication events and, consequently, could share similar ligand binding preferences. This could be consistent with our MD simulation experiments that show stable interactions of the two related diamines in both receptors. Moreover, taking into account that besides the Asp_3.32_/Asp_5.43_ pair, all other negatively charged binding pocket residues are also conserved in the TAAR6 and TAAR8 sequences (Fig 2 and S1 Fig). It is reasonable to assume that a common molecular mechanism for PUT and CAD recognition is shared by the mammalian orthologs here identified.

## Discussion

Death’s distinctive smell, characterized among other chemicals by the volatiles diamines PUT and CAD, constitutes an important signal related to risk avoidance, social cues and feeding behaviors which are pivotal for surviving. PUT and CAD belong to the biogenic amine group of naturally occurring compounds found in the whole animal world from bacteria to mammals, including key intracellular signaling molecules with powerful physiological effects such as histamine, serotonin, dopamine and adrenaline [45]. But unlike these well-studied neurotransmitters, the molecular basis and physiological actions of these ‘necromones’ is still largely unknown. Fortunately, there is indication that zebrafish TAAR13c constitutes a diamine sensor that manifests selectivity for odd chain diamines, including CAD. With this knowledge, we explored the sequence-structure relation of TAARs from different organisms and propose the human TAAR6 and TAAR8, and possibly their mammalian orthologs, as the cognate receptors for these compounds. This finding is supported by the analysis of structure-informed sequence alignments of close related aminergic GPCRs, revealing a conserved tandem of negatively charged aspartates in the ligand binding cavity of teleost TAAR13c and mammalian TAAR6 and TAAR8, which are likely to be involved in diamine recognition. Structural models of these receptors based on 3D structures of the ADRB2 in different conformational states, together with molecular docking and MD simulations, sustain this hypothesis, showing feasible interactions between the negatively charged aspartates Asp_3.32_ (zTAAR13cD112; hTAAR6 D112; hTAAR8 D111) and Asp_5.42/5.43_ (zTAAR13cD202; hTAAR6 D202; hTAAR8 D201) with diamine moieties of PUT and CAD. The observation that both TAAR6 and TAAR8 could bind these similar molecules is not surprising, in view of the well-known ligand promiscuity among closely related GPCRs (e.g. both adrenaline and noradrenaline display high affinity for alpha-adrenergic ADRA1 and ADRA2 receptors). Unfortunately, our theoretical approach does not allow to predict the binding affinities for these similar binders (C4 vs. C5 alkyl chain lengths), in either TAAR6 or TAAR8. However, since the interactions between Asp_3.32/5.43_ (-COO^-^) and PUT/CAD (-NH3^+^) were more stable in the active-like complexes, following a similar trend as that observed for the CAD binding to the zTAAR13c, we hypothesize that both ligands show a preference for the activated state of the receptors and, consequently, could behave as agonists.

Taking into account that the odor mortis constitutes a primordial class of chemical signal linked to survival, the two-aspartate signature was searched amongst TAARs of other jawed vertebrates. Teleosts (bony fishes) are characterized by a great expansion of TAAR genes (including TAAR13c and TAAR13d) related to the important roles of solubilized polyamines for chemical communication in water environments [3]. Conversely, no identifiable TM3 and TM5 negatively charged signature was found in sauropsids (birds and reptiles) or amphibian lineages, characterized by small number of TAARs, but with large numbers of vomeronasal and taste receptor repertoires [42]. This great amount of variation in chemosensory receptors within organisms, has been linked to a model of birth-and-death evolution, related to living environments [43, 46]. Thus, specific ecological conditions [47], lineage-specific specialization [48] and morphological or physiological adaptations [49] among other factors, could lead to different sensory abilities to detect the PUT and CAD polyamines in these species.

In mammals, the tertiary amine-detecting TAARs display higher rates of gene duplications, which suggest they may have played important roles in terrestrial adaptations. Likewise, the high conservation of the negatively charged Asp_3.32_/Asp_5.43_ tandem in TAAR6 and TAAR8 therian sequences seems to provide chemosensory sensitivity to diamines like PUT and CAD in most of terrestrial mammals. Nonetheless, this signature is missing in the non-terrestrial aquatic dolphins and whales, characterized in general by having small number functional chemosensory receptors [50] and in some carnivores like dog [51]. In the latter case, the notable loss of functional TAARs seems to be compensated by a strong evolution of ORs genes (> 800) which almost double the human repertoire [52]. It is known that OR-expressing neurons may also function as detectors of trace amines in the olfactory epithelium [53]. Thus, from this perspective, the rapid evolutionary diversification according to environmental adaptations makes it possible that recognition of PUT and CAD in vertebrates lacking TAAR6 and TAAR8 functional genes, could be undertaken by other chemosensory receptors which may have developed a dication binding site. In any event, these primordial class of chemical signals linked to the survival of many organisms deserve further studies. We hope this work helps provide insight into two scarcely studied human receptors with unknown pharmacology and contribute to the understanding of the mechanism of action of PUT and CAD which may be useful in pharmacological applications and other industrial purposes.

## Methods

### Protein sequence retrieval and alignment

The human TAAR6 (NP_778237.1) and TAAR8 (NP_444508.1) were used as queries to search for homologues using protein-protein blast (blastp) sequence similarity searches (http://www.ncbi.nlm.nih.gov/blast). Twenty-six TAAR6 and TAAR8 mammalian orthologs (including humans) were aligned with ClustalW, using the GPCRtm substitution matrix [54] (see S1 Fig). An additional MSA was constructed with a selection of TAAR6, TAAR8, TAAR13c and TAAR13d sequences and related aminergic receptors with known 3D-structures. This MSA was manually curated in order to satisfy the structural correspondence between conserved sequence motifs in class A GPCRs, including the disulfide bridge between TM3 and ECL2 [29] and a single residue gap in TM5 [25] (see Fig 1). Approximate divergence times between species were estimated with TimeTree [55].

### Homology modeling

MODELLER v9.12 [56] was used for the construction of hTAAR6, hTAAR8 and zTAAR13c three-dimensional (3D) models using the crystal structures of the closed related ADRB2 as templates (reference MSA on Fig 1). Only non-conserved N-terminal (amino acids 1–20), C-terminal (amino acids 329–345) and ICL3 (amino acids 226–251) regions were excluded for the modeling protocol. One hundred models were generated for each receptor in the active-like (template PDB ID: 3P0G, [32]) and inactive-like conformations (template PDB ID: 2RH1, [31]) (see S2 Fig). The resulting models were evaluated stereochemically with ProSA and PROCHECK (S1 Table). The best evaluated structures were selected for further refinement of loop regions through a MD simulated annealing (SA) protocol. For this purpose, the backbone residues of the TM helices were constrained and the conformation of ECLs and ICLs were optimized in 20 simulated annealing cycles of heating up to 700 K and slowly cooling down to 300 K in successive 10 K, 100 ps steps, followed by an energy minimization with the AMBER ff99SB force field [57].

### Molecular docking

PUT and CAD were docked into the hTAAR6 and hTAAR8 models using the Molecular Operating Environment (MOE) [58]. The Site Finder application in MOE was employed to localize the binding cavities from the 3D atomic coordinates of the molecular models and 100 conformations per ligand were generated by the stochastic conformation search method. One hundred flexible docking solutions were produced by the triangle matcher algorithm into the active site of the receptor structures (additional details on S2 Table). Top-ranking solutions were visually inspected and the high score conformations in which the protonated amines form ionic interactions with Asp_3.32_ and Asp_5.43_ were energy minimized (S4 Fig). A similar protocol was employed for docking CAD to its cognate receptor zTAAR13c (S2 Table and S5 Fig). The selected binding complexes were further studied in explicit membrane MD simulations with the GROMACS MD simulation package.

### Molecular dynamics simulations

MD simulations were performed using GROMACS v5.0.7. Ten molecular systems: hTAAR6_active-like_/PUT; hTAAR6_active-like_/CAD; hTAAR6_inactive-like_/PUT; hTAAR6_inactive-like_/CAD; hTAAR8_active-like_/PUT; hTAAR8_active-like_/CAD; hTAAR8_inactive-like_/PUT; hTAAR8_inactive-like_/CAD; zTAAR13c_active-like_/CAD and zTAAR13c_inactive-like_/CAD were embedded in pre-equilibrated lipid bilayers containing 1-palmitoyl-2-oleoyl-sn-glycero-3-phosphatidylcholine (POPC), water molecules (TIP3P) and monoatomic Na^+^ and Cl^-^ ions (0.2 M), with its long axis perpendicular to the membrane interface (additional information on S3 Table). Taking into account that agonists alone are not able to preserve a fully active conformation of the receptor in the absence of the G protein [37], in our simulations, the active-like models were further stabilized by the inclusion of the G protein mimic nanobody particle towards the cytoplasmic region [32] (shown in S2 and S6 Figs). MD systems were subject to a 1000 steps of energy minimization, followed by 20.0 ns of gradual relaxation of positional restraints in protein backbone coordinates before the production phase in order to hydrate the receptor cavities and allow lipids to pack around the protein. After equilibration, 1 μs unrestrained MD trajectories were generated at a constant temperature of 300 K using separate v-rescale thermostats for the receptor, ligand, lipids and solvent molecules. A time step of 2.0 fs was used for the integration of equations of motions. All bonds and angles were kept frozen using the LINCS algorithms. Lennard-Jones interactions were computed using a cutoff of 10 Å, and the electrostatic interactions were treated using PME with the same real-space cutoff under periodic boundary conditions (PBC). The AMBER ff99SB force field was selected for the protein and the parameters described by Berger and co-workers was used for the lipids [59]. PUT and CAD parameters were obtained from the general Amber force field (GAFF) and HF/6-31G*-derived RESP atomic charges.

## Supporting information

S1 Fig Sequence comparison of TAAR6 and TAAR8 mammalian orthologs. MSA of 26 selected TAAR6 and TAAR8 protein sequences from mammals. Predicted TM helices boundaries are represented at the top of the alignment. Conserved positions are highlighted in grayscale according to sequence conservation. Highly conserved residues in the class A GPCR family are indicated by the Ballesteros-Weinstein numbering (X.50 of helix X), as well as the negatively charged residues (red labels) lining the ligand binding cavity according to the hTAAR6 and hTAAR8 molecular models (see Fig 2). Non-conserved N-and C- terminal regions are omitted from the figure. Two-letter acronyms and NCBI sequence accession numbers for each species correspond to: *md* (*Monodelphis domestica*; XP_001380535.1, XP_001380502.1), *tm* (*Trichechus manatus*; XP_012410597.1, XP_004368972.1), *la* (*Loxodonta Africana*; XP_003404135.1, XP_003404152.1), *bt* (*Bous Taurus*; XP_002690274.1), *cd* (*Camelus dromedarius*; XP_010986976.1, XP_010986975.1), *ss* (*Sus scrofa*; XP_001926423.1, XP_001926072.1), *ec* (*Equus caballus*; XP_001503412.1, XP_014591123.1), *um* (*Ursus maritimus*; XP_008689453.1, XP_008689314.1), *fc* (*Felis catus*; XP_003986612.1), *rn* (*Rattus norvegicus*; NP_783174.1, NP_783189.1), *mm* (*Mus musculus*; NP_001010828.1, NP_001010837.1), *pa* (*Pongo abelii*; XP_009240535.1, XP_009240534.1), *gg* (*Gorilla gorilla*; XP_004065396.2, XP_018872342.1) and *hs* (*Homo sapiens*; NP_778237.1, NP_444508.1).
(TIF)

S2 Fig Structural features of the human TAAR6 and TAAR8 homology models. (A) 3D-coordinates superimposition of the hTAAR6 and hTAAR8 molecular models in the “active-like” (template PDB ID: 3P0G, green-orange ribbons) and “inactive-like” (template PDB ID: 2RH1, light-grey ribbons) conformations (see Methods). Lateral (top) and extracellular view (bottom) of the best evaluated models by ProSA and PROCHECK (see S1 Table). Residues of the ligand binding pocket are shown in sticks (av. RMSD < 2Å in all models). (B) Most important differences are located at the cytoplasmic G protein-coupling domain (outward displacement of TM5 ~5Å, TM6 ~10Å and moderate displacement of TM7 and helix 8 ~3Å towards the receptor core, orange ribbon). These structural changes in the active-like conformations allows the coupling with the G-Protein or a Nanobody particle (Nb80, purple ribbon).
(TIF)

S3 Fig The orthosteric ligand-binding pocket of zebrafish TAAR13c. Surface representation of the molecular models of zTAAR13c in the active- (A) and inactive-like (B) conformations. Extracellular view of the ligand binding cavities with molecular surfaces colored by the electrostatic potential calculated using the program APBS with nonlinear Poisson-Boltzmann equation and contoured at ±10 kT/e (negatively and positively charged surface areas in red and blue, respectively). Main residues contributing to the electronegative potential of the binding pocket are represented in sticks (Ballesteros-Weinstein numbering scheme in parenthesis). Calculated distances between carboxyl moieties of Asp_3.32_ and Asp_5.42_ are shown for each molecular structure (yellow dashed lines). Protein backbones are shown in cylinders except ECL2 conformations (omitted for clarity).
(TIF)

S4 Fig Molecular interactions of PUT and CAD with human TAAR6 and TAAR8. Selected docking complexes of PUT and CAD to the human TAAR6 (light-gray) and TAAR8 (blue ribbons) in different conformational states: [hTAAR6_active-like_/hTAAR8_active-like_; template PDB ID: 3P0G] and [hTAAR6_inactive-like_/hTAAR8_inactive-like_; template PDB ID: 2RH1]. Figure shows the extracellular view of the binding cavity for each receptor (cartoon representation) and residues within 3.0 Å of the diamine ligands (D_3.32_, C_3.36_, L/S_5.46_, D_5.43_, W_6.48_, Y_6.51_, T/S_6.52_ and Y_7.43_, in sticks). Receptors are oriented in the same direction of bottom panel A on S2 Fig.
(TIF)

S5 Fig Molecular interactions of CAD with zebrafish TAAR13c. Molecular docking complexes of cadaverine (in orange sticks) to the active-like zTAAR13c model (A, in grey ribbons) and inactive-like (B, in blue ribbons). Figure shows the extracellular view of the binding cavity in the molecular structures with residues of the receptor at a distance < 3.5Å from the ligand in sticks (numbered according to Ballesteros-Weinstein scheme). Predicted (ligand–receptor) ionic interactions are shown in yellow dashed lines.
(TIF)

S6 Fig Molecular dynamics (MD) simulation systems. Lateral view of representative molecular systems corresponding to (A) the “active-like” and, (B) the “inactive-like” TAAR conformations complexed with the PUT and CAD ligands. Ligand-receptor complexes were embedded in a lipid bilayer (yellow vdW spheres) with explicit solvent (light blue) and counterions (small spheres) (details on S3 Table). MD simulations were performed with GROMACS (see Methods).
(TIF)

S7 Fig Stability of interactions between PUT and CAD with human TAAR6 and TAAR8. Root mean square deviation (RMSD, in angstrom Å on the y-axis) of the diamine ligands complexed to active- and inactive-like conformations of human TAAR6 and TAAR8 during (1μs, on the x-axis) of unrestrained MD simulations. The stability of the binding is confirmed by the small fluctuations of PUT/CAD coordinates, in particular for the active-like structures (av. RMSD_ligand_ ~2.0 Å on top). The larger fluctuations observed on the inactive-like complexes (bottom) correspond to spin movements of the ligands inside the binding pocket (see Fig 4).
(TIF)

S8 Fig Stability of interactions between CAD and zebrafish TAAR13c. Root mean square deviation (RMSD, in angstrom Å) of cadaverine in the active- (A) and inactive-like (B) zTAAR13c structures during 1μs of unrestrained MD simulations. The time evolution of intermolecular distances between N1/N2 atoms of the ligand and Asp_3.32_ (in red) and Asp_5.43_ (blue) carboxyl groups in the respective simulated systems are displayed in (C and D). The stability of the binding is confirmed by the small fluctuations of the ligand coordinates, in particular for the active-like complex (A, C). The larger fluctuation of the ligand observed on the inactive-like complex (B and D) correlates with variation in the Asp_5.43_(-COO^-^)-CAD(-NH3^+^) distance inside the binding pocket.
(TIF)

S9 Fig Effect of PUT and CAD on the ‘transmission switch’ region in the TAAR6 and TAAR8. Distribution of the positions of the Cβ atoms (green and red dots) corresponding to the L/V_3.40_, P_5.50_ and F_6.44_ residues (in sticks) during 1.0 μs of unbiased MD simulations of the human TAAR6 (light-gray) and TAAR8 (blue) in active- and inactive-like conformational states. For comparison purposes the hTAAR6/hTAAR8 molecular models were superimposed to the ADRB2 crystallographic structures in agonist bound active (PDB ID:3SN6; green sticks) and inverse agonist bound inactive conformation (PDB ID:2RH1; red sticks). Numbers in parentheses correspond to the average distance between the Cβ positions of 100 evenly spaced snapshots extracted from the MD simulation and the centroid of those positions.
(TIF)

S1 Table Homology modeling and structure validation of TAARs. Ramachandran plot summaries of the selected hTAAR6, hTAAR8 and zTAAR13c models in the ‘active-like’ and ‘inactive-like’ conformations and its respective templates (PDB IDs: 3P0G and 2RH1) obtained from PROCHECK program [60]. Accuracy of the generated models was also evaluated and compared with the crystallographic references using ProSA-web [61]. The resulting 3D-coordinates from the refinement of loop regions through a MD SA protocol (see Methods) were also calculated. The overall statistics of structure quality indicate that structural templates and generated models have similar values.
(DOCX)

S2 Table Summary of molecular docking results of PUT and CAD with TAARs. Selected docking solutions of PUT/CAD to the hTAAR6/hTAAR8 molecular models and CAD to the zTAAR13c in different conformational states. Flexible docking of the ligands was performed with MOE v2013.08 using the ‘active-like’ and ‘inactive-like’ conformations of the modeled receptors. The ligands' 2D chemical structures were drawn in ChemDraw (v16.0, PerkinElmer) and a stochastic conformational search was performed in order to generate 3D conformations. The number of conformations was limited to a maximum of 100 per ligand and duplicates conformations (RMSD < 0.25 Å) were removed. The binding site region was defined using the site points created by MOE's Site Finder application and included residues in contact with co-crystallized ligands found in the PDB structures of biogenic amine receptors 5-HT1BR (PDB ID: 4IAR), ADRB2 (2RH1, 3P0G), D3R (3PBL), H1R (3RZE). Molecular docking protocol employed the triangle matcher placement method and the London dG scoring function. Each binding pose was then minimized and rescored with the GBVI/WSA ΔG scoring function [62]. Modeled receptors were parameterized using Amber ff99SB [63]. The ligand bonded parameters were obtained with 2D extended Hückel theory [64]. VdW parameters were derived from GAFF [65] and the charges from bond charge increments according to the AMBER10:EHT force field option in MOE. Docking poses were selected on basis of the interaction distance among the Cγ atoms of Asp_3.32/5.43_ and PUT/CAD (N1, N2) amine nitrogen’s with lower docking score energies.
(DOCX)

S3 Table MD simulations performed in this study. Ten molecular systems: hTAAR6_active-like_/PUT, hTAAR6_active-like_/CAD, hTAAR8_active-like_/PUT, hTAAR8_active-like_/CAD, zTAAR13c_active-like_/CAD, hTAAR6_inactive-like_/PUT, hTAAR6_inactive-like_/CAD, hTAAR8_inactive-like_/PUT, hTAAR8_inactive-like_/CAD and zTAAR13c_inactive-like_/CAD were embedded in pre-equilibrated lipid bilayers containing 1-palmitoyl-2-oleoyl-sn-glycero-3-phosphatidylcholine (POPC), water molecules (TIP3P) and monoatomic Na+ and Cl- ions (0.2 M). All distances and RMSD values are shown in Angstroms (Å). *Reference experimental values DH-H POPC (303K): 37,0 [66].
(DOCX)
