# Supplementary figures and images for: Identifying human diamine sensors for death related putrescine and cadaverine molecules
Source: PLoS Comput Biol. 2018 Jan 11;14(1):e1005945. doi: 10.1371/journal.pcbi.1005945 (PMC5783396; doi:10.1371/journal.pcbi.1005945)

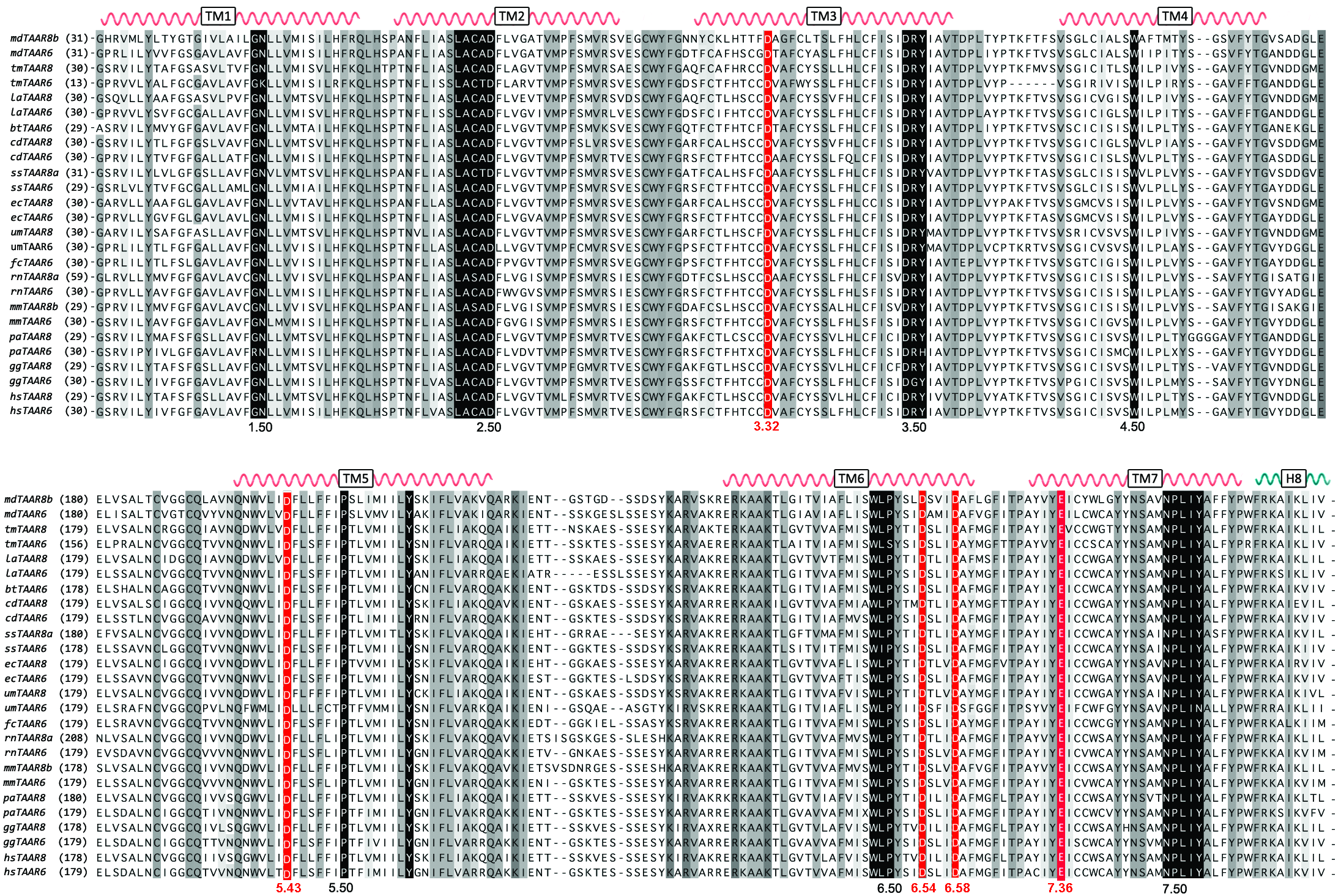

Supplement: S1 Fig — MSA of 26 selected TAAR6 and TAAR8 protein sequences from mammals. Predicted TM helices boundaries are represented at the top of the alignment. Conserved positions are highlighted in grayscale according to sequence conservation. Highly conserved residues in the class A GPCR family are indicated by the Ballesteros-Weinstein numbering (X.50 of helix X), as well as the negatively charged residues (red labels) lining the ligand binding cavity according to the hTAAR6 and hTAAR8 molecular models (see Fig 2). Non-conserved N-and C- terminal regions are omitted from the figure. Two-letter acronyms and NCBI sequence accession numbers for each species correspond to: md (Monodelphis domestica; XP_001380535.1, XP_001380502.1), tm (Trichechus manatus; XP_012410597.1, XP_004368972.1), la (Loxodonta Africana; XP_003404135.1, XP_003404152.1), bt (Bous Taurus; XP_002690274.1), cd (Camelus dromedarius; XP_010986976.1, XP_010986975.1), ss (Sus scrofa; XP_001926423.1, XP_001926072.1), ec (Equus caballus; XP_001503412.1, XP_014591123.1), um (Ursus maritimus; XP_008689453.1, XP_008689314.1), fc (Felis catus; XP_003986612.1), rn (Rattus norvegicus; NP_783174.1, NP_783189.1), mm (Mus musculus; NP_001010828.1, NP_001010837.1), pa (Pongo abelii; XP_009240535.1, XP_009240534.1), gg (Gorilla gorilla; XP_004065396.2, XP_018872342.1) and hs (Homo sapiens; NP_778237.1, NP_444508.1). (TIF) [file pcbi.1005945.s001.tif]

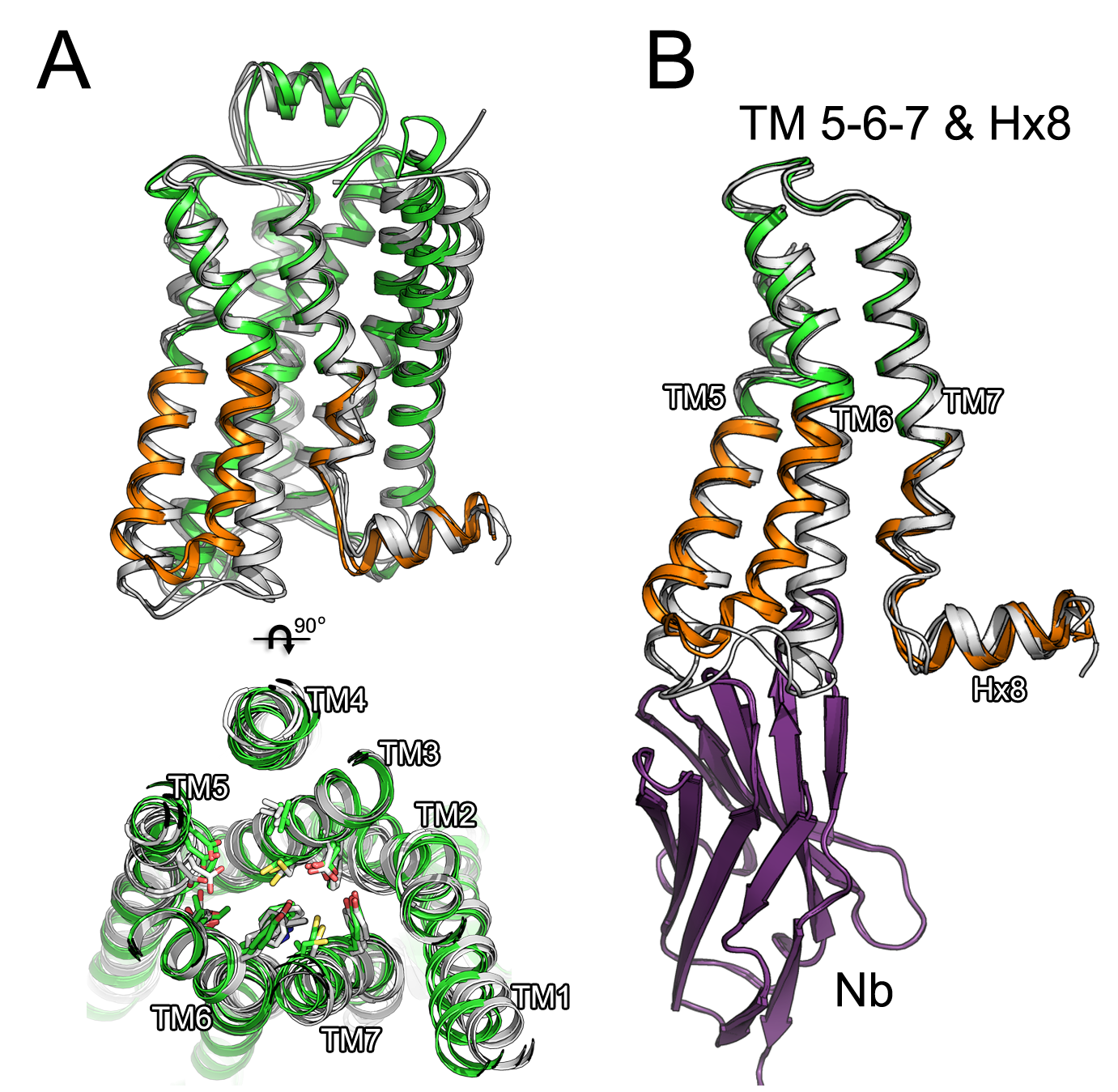

Supplement: S2 Fig — (A) 3D-coordinates superimposition of the hTAAR6 and hTAAR8 molecular models in the “active-like” (template PDB ID: 3P0G, green-orange ribbons) and “inactive-like” (template PDB ID: 2RH1, light-grey ribbons) conformations (see Methods). Lateral (top) and extracellular view (bottom) of the best evaluated models by ProSA and PROCHECK (see S1 Table). Residues of the ligand binding pocket are shown in sticks (av. RMSD < 2Å in all models). (B) Most important differences are located at the cytoplasmic G protein-coupling domain (outward displacement of TM5 ~5Å, TM6 ~10Å and moderate displacement of TM7 and helix 8 ~3Å towards the receptor core, orange ribbon). These structural changes in the active-like conformations allows the coupling with the G-Protein or a Nanobody particle (Nb80, purple ribbon). (TIF) [file pcbi.1005945.s002.tif]

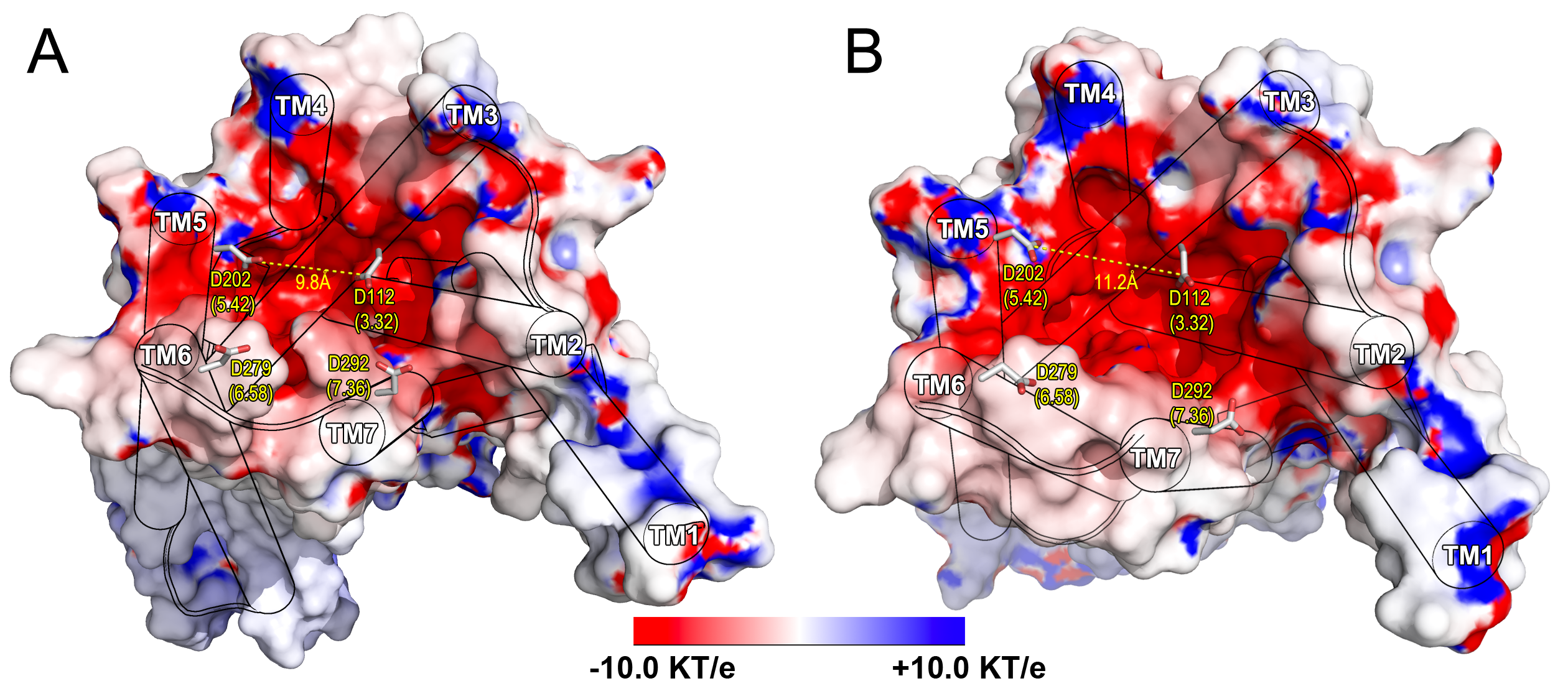

Supplement: S3 Fig — Surface representation of the molecular models of zTAAR13c in the active- (A) and inactive-like (B) conformations. Extracellular view of the ligand binding cavities with molecular surfaces colored by the electrostatic potential calculated using the program APBS with nonlinear Poisson-Boltzmann equation and contoured at ±10 kT/e (negatively and positively charged surface areas in red and blue, respectively). Main residues contributing to the electronegative potential of the binding pocket are represented in sticks (Ballesteros-Weinstein numbering scheme in parenthesis). Calculated distances between carboxyl moieties of Asp3.32 and Asp5.42 are shown for each molecular structure (yellow dashed lines). Protein backbones are shown in cylinders except ECL2 conformations (omitted for clarity). (TIF) [file pcbi.1005945.s003.tif]

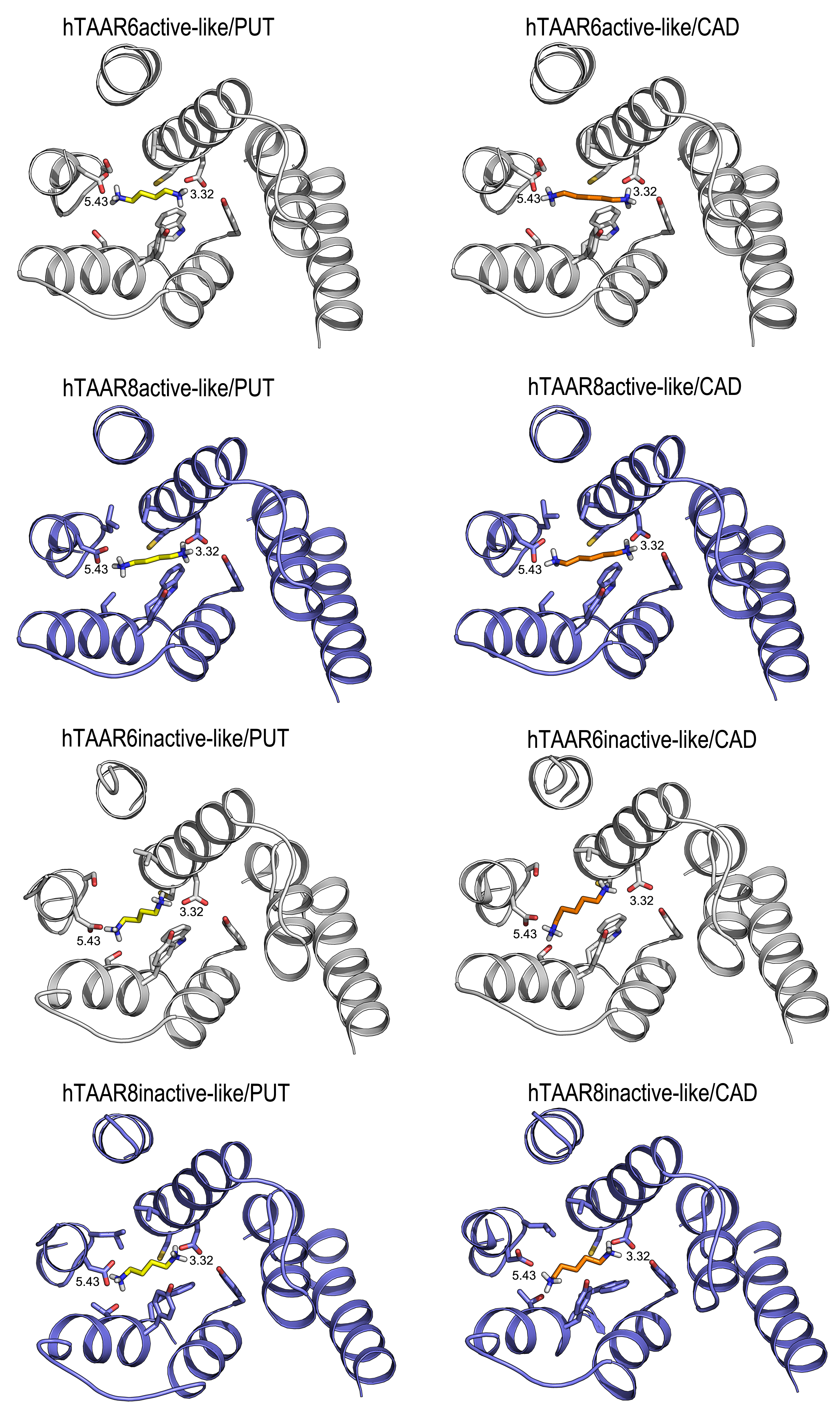

Supplement: S4 Fig — Selected docking complexes of PUT and CAD to the human TAAR6 (light-gray) and TAAR8 (blue ribbons) in different conformational states: [hTAAR6active-like/hTAAR8active-like; template PDB ID: 3P0G] and [hTAAR6inactive-like/hTAAR8inactive-like; template PDB ID: 2RH1]. Figure shows the extracellular view of the binding cavity for each receptor (cartoon representation) and residues within 3.0 Å of the diamine ligands (D3.32, C3.36, L/S5.46, D5.43, W6.48, Y6.51, T/S6.52 and Y7.43, in sticks). Receptors are oriented in the same direction of bottom panel A on S2 Fig. (TIF) [file pcbi.1005945.s004.tif]

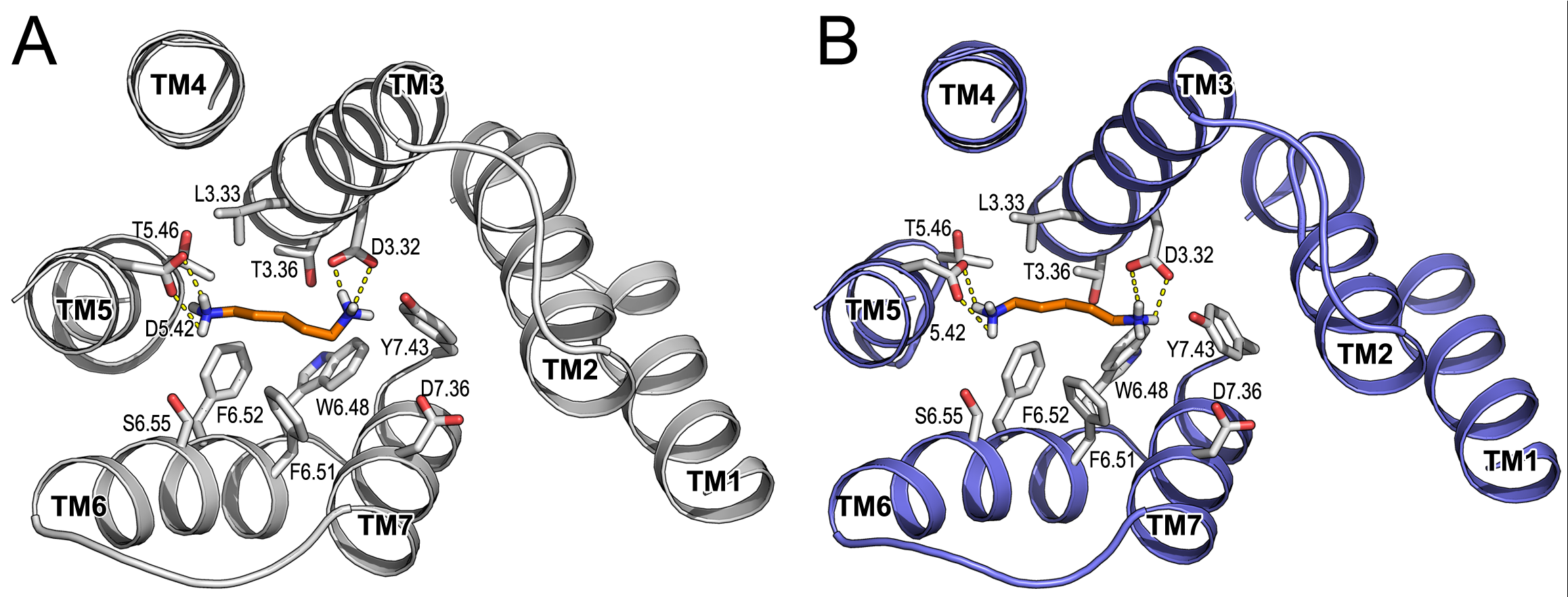

Supplement: S5 Fig — Molecular docking complexes of cadaverine (in orange sticks) to the active-like zTAAR13c model (A, in grey ribbons) and inactive-like (B, in blue ribbons). Figure shows the extracellular view of the binding cavity in the molecular structures with residues of the receptor at a distance < 3.5Å from the ligand in sticks (numbered according to Ballesteros-Weinstein scheme). Predicted (ligand–receptor) ionic interactions are shown in yellow dashed lines. (TIF) [file pcbi.1005945.s005.tif]

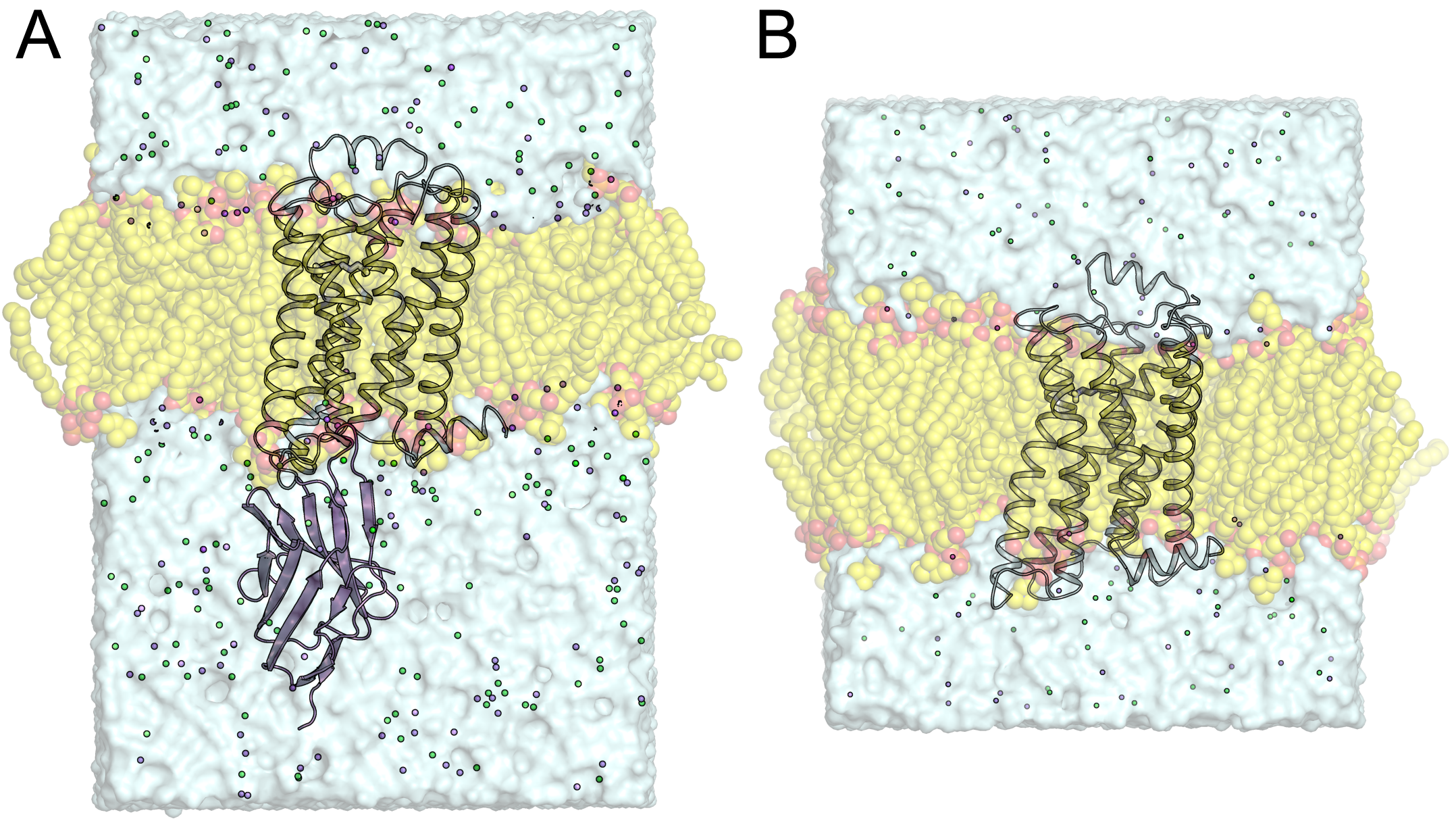

Supplement: S6 Fig — Lateral view of representative molecular systems corresponding to (A) the “active-like” and, (B) the “inactive-like” TAAR conformations complexed with the PUT and CAD ligands. Ligand-receptor complexes were embedded in a lipid bilayer (yellow vdW spheres) with explicit solvent (light blue) and counterions (small spheres) (details on S3 Table). MD simulations were performed with GROMACS (see Methods). (TIF) [file pcbi.1005945.s006.tif]

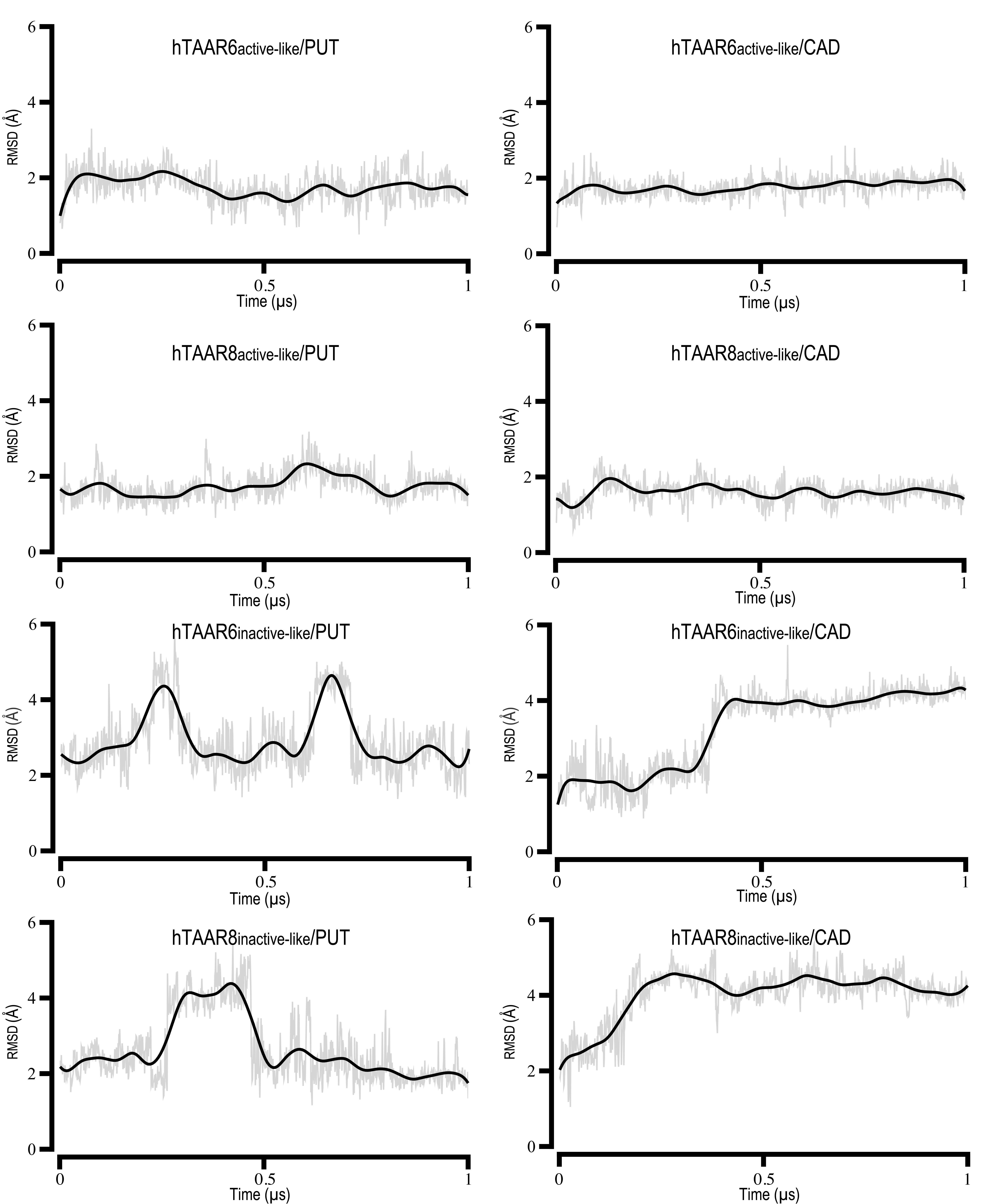

Supplement: S7 Fig — Root mean square deviation (RMSD, in angstrom Å on the y-axis) of the diamine ligands complexed to active- and inactive-like conformations of human TAAR6 and TAAR8 during (1μs, on the x-axis) of unrestrained MD simulations. The stability of the binding is confirmed by the small fluctuations of PUT/CAD coordinates, in particular for the active-like structures (av. RMSDligand ~2.0 Å on top). The larger fluctuations observed on the inactive-like complexes (bottom) correspond to spin movements of the ligands inside the binding pocket (see Fig 4). (TIF) [file pcbi.1005945.s007.tif]

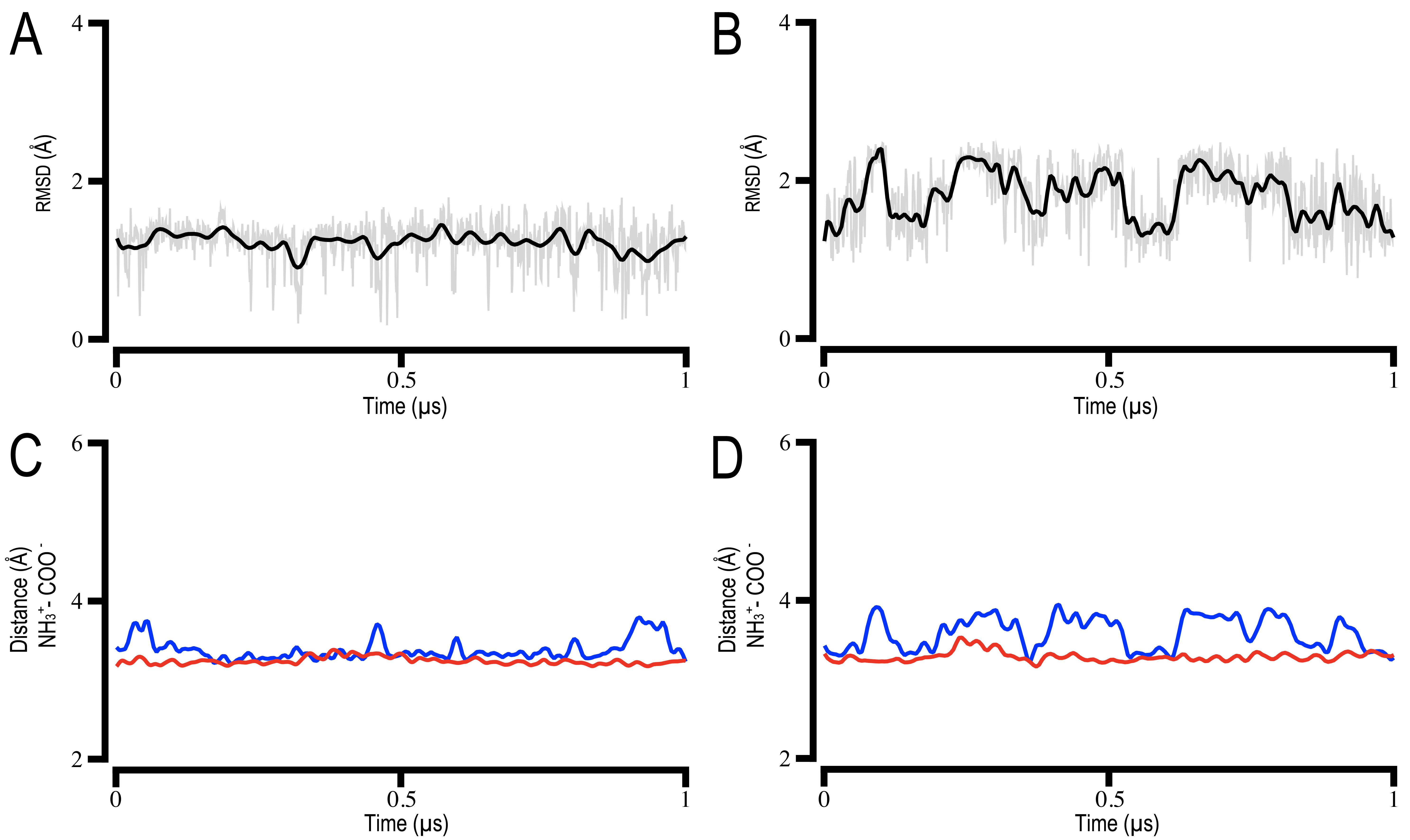

Supplement: S8 Fig — Root mean square deviation (RMSD, in angstrom Å) of cadaverine in the active- (A) and inactive-like (B) zTAAR13c structures during 1μs of unrestrained MD simulations. The time evolution of intermolecular distances between N1/N2 atoms of the ligand and Asp3.32 (in red) and Asp5.43 (blue) carboxyl groups in the respective simulated systems are displayed in (C and D). The stability of the binding is confirmed by the small fluctuations of the ligand coordinates, in particular for the active-like complex (A, C). The larger fluctuation of the ligand observed on the inactive-like complex (B and D) correlates with variation in the Asp5.43(-COO-)-CAD(-NH3+) distance inside the binding pocket. (TIF) [file pcbi.1005945.s008.tif]

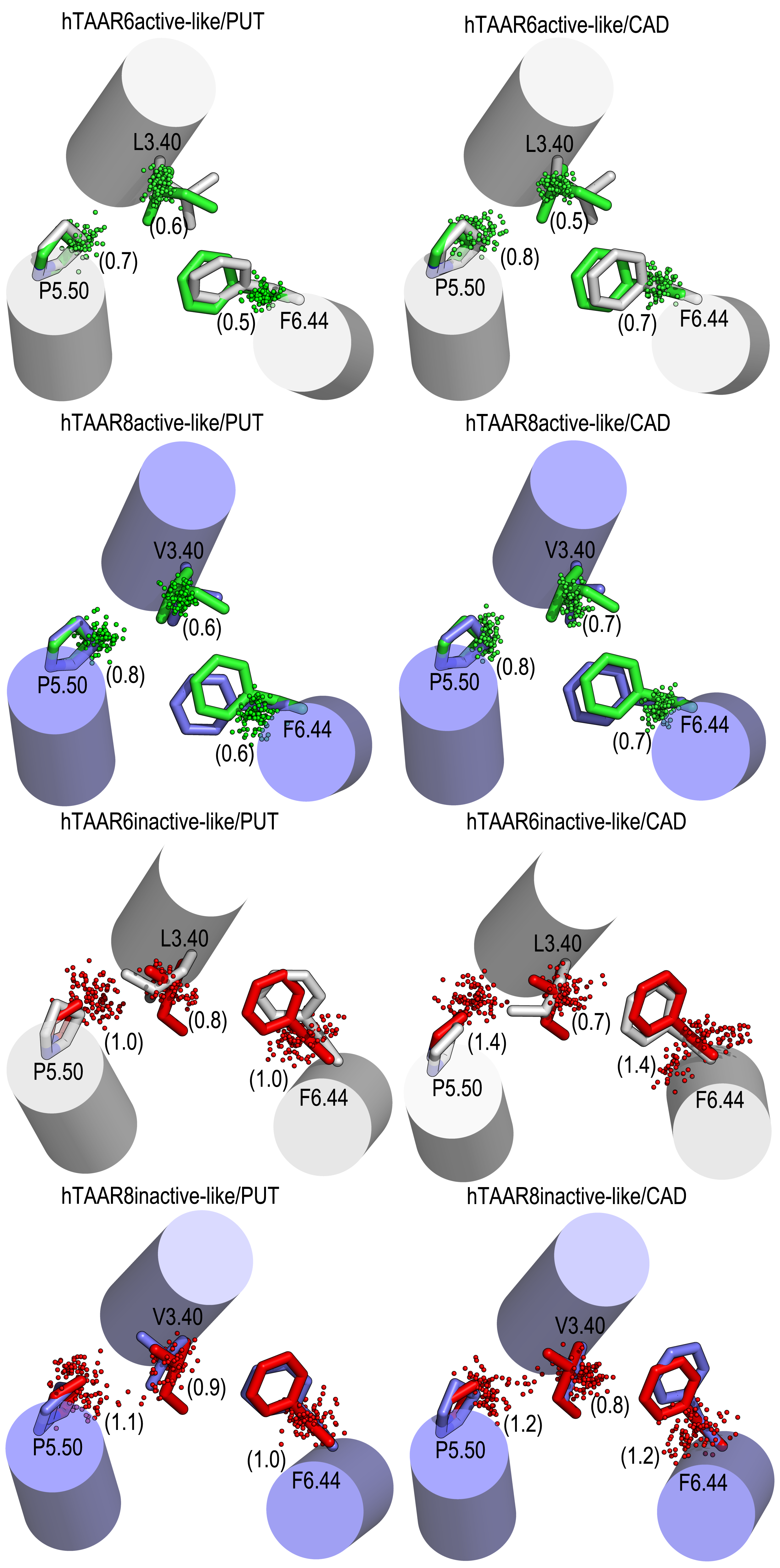

Supplement: S9 Fig — Distribution of the positions of the Cβ atoms (green and red dots) corresponding to the L/V3.40, P5.50 and F6.44 residues (in sticks) during 1.0 μs of unbiased MD simulations of the human TAAR6 (light-gray) and TAAR8 (blue) in active- and inactive-like conformational states. For comparison purposes the hTAAR6/hTAAR8 molecular models were superimposed to the ADRB2 crystallographic structures in agonist bound active (PDB ID:3SN6; green sticks) and inverse agonist bound inactive conformation (PDB ID:2RH1; red sticks). Numbers in parentheses correspond to the average distance between the Cβ positions of 100 evenly spaced snapshots extracted from the MD simulation and the centroid of those positions. (TIF) [file pcbi.1005945.s009.tif]
