# Supplementary material for: Identifying human diamine sensors for death related putrescine and cadaverine molecules
Source: PLoS Comput Biol. 2018 Jan 11;14(1):e1005945. doi: 10.1371/journal.pcbi.1005945 (PMC5783396; doi:10.1371/journal.pcbi.1005945)
Supplement: S1 Table — Ramachandran plot summaries of the selected hTAAR6, hTAAR8 and zTAAR13c models in the ‘active-like’ and ‘inactive-like’ conformations and its respective templates (PDB IDs: 3P0G and 2RH1) obtained from PROCHECK program [60]. Accuracy of the generated models was also evaluated and compared with the crystallographic references using ProSA-web [61]. The resulting 3D-coordinates from the refinement of loop regions through a MD SA protocol (see Methods) were also calculated. The overall statistics of structure quality indicate that structural templates and generated models have similar values. (DOCX) [file pcbi.1005945.s010.docx]

| GPCR | 3D-Coordinates | Ramachandran Plot Summary from Procheck | | | | Overall model quality  ProSA-web |
| --- | --- | --- | --- | --- | --- | --- |
|  |  | Most favoured  regions | Additionally  allowed regions | Generously  allowed regions | Disallowed  regions | Z-Score |
| hADRB2 | PDB_ID_ 3P0G | 88,1% | 11,9% | 0,0% | 0,0% | -1,88 |
|  | PDB_ID_ 2RH1 | 94,2% | 5,4% | 0,0% | 0,0% | -2,52 |
| hTAAR6_active-like_ | Selected Model | 92,1% | 7,9% | 0,0% | 0,0% | -1,51 |
|  | SA-loop refinement | 93,5% | 6,5% | 0,0% | 0,0% | -1,77 |
| hTAAR6_inactive-like_ | Selected Model | 89,6% | 9,2% | 0,8% | 0,4% | -2,17 |
|  | SA-loop refinement | 92,9% | 6,3% | 0,8% | 0,0% | -2,34 |
| hTAAR8_active-like_ | Selected Model | 89,8% | 9,8% | 0,4% | 0,0% | -1,60 |
|  | SA-loop refinement | 91,2% | 8,8% | 0,0% | 0,0% | -1,84 |
| hTAAR8_inactive-like_ | Selected Model | 90,5% | 8,7% | 0,4% | 0,4% | -2,75 |
|  | SA-loop refinement | 93,3% | 6,7% | 0,0% | 0,0% | -2,93 |
| zTAAR13c_active-like_ | Selected Model | 94,1% | 5,5% | 0,4% | 0,0% | -2,57 |
|  | SA-loop refinement | 95,1% | 4,9% | 0,0% | 0,0% | -2,61 |
| zTAAR13c_inactive-like_ | Selected Model | 95,3% | 4,3% | 0,4% | 0,0% | -2,66 |
|  | SA-loop refinement | 96,5% | 2,8% | 0,8% | 0,0% | -2.71 |
