# Supplementary material for: Identifying human diamine sensors for death related putrescine and cadaverine molecules
Source: PLoS Comput Biol. 2018 Jan 11;14(1):e1005945. doi: 10.1371/journal.pcbi.1005945 (PMC5783396; doi:10.1371/journal.pcbi.1005945)
Supplement: S2 Table — Selected docking solutions of PUT/CAD to the hTAAR6/hTAAR8 molecular models and CAD to the zTAAR13c in different conformational states. Flexible docking of the ligands was performed with MOE v2013.08 using the ‘active-like’ and ‘inactive-like’ conformations of the modeled receptors. The ligands' 2D chemical structures were drawn in ChemDraw (v16.0, PerkinElmer) and a stochastic conformational search was performed in order to generate 3D conformations. The number of conformations was limited to a maximum of 100 per ligand and duplicates conformations (RMSD < 0.25 Å) were removed. The binding site region was defined using the site points created by MOE's Site Finder application and included residues in contact with co-crystallized ligands found in the PDB structures of biogenic amine receptors 5-HT1BR (PDB ID: 4IAR), ADRB2 (2RH1, 3P0G), D3R (3PBL), H1R (3RZE). Molecular docking protocol employed the triangle matcher placement method and the London dG scoring function. Each binding pose was then minimized and rescored with the GBVI/WSA ΔG scoring function [62]. Modeled receptors were parameterized using Amber ff99SB [63]. The ligand bonded parameters were obtained with 2D extended Hückel theory [64]. VdW parameters were derived from GAFF [65] and the charges from bond charge increments according to the AMBER10:EHT force field option in MOE. Docking poses were selected on basis of the interaction distance among the Cγ atoms of Asp3.32/5.43 and PUT/CAD (N1, N2) amine nitrogen’s with lower docking score energies. (DOCX) [file pcbi.1005945.s011.docx]

| Receptor type | Ligand | Docking Rank | London dG scoring (kcal/mol) | GBVI/WSA Δ*G* scoring (kcal/mol) | Distance  Asp_3.32_ Cγ - Lig N_1_ (Å) | Distance  Asp_5.43_ Cγ - Lig N_2_ (Å) |
| --- | --- | --- | --- | --- | --- | --- |
| hTAAR6_active-like_ | PUT | 4 of 100 | -9,13 | -4,20 | 3,4 | 3,6 |
|  | CAD | 2 of 100 | -10,13 | -4,54 | 3,2 | 3,5 |
| hTAAR6_inactive-like_ | PUT | 3 of 100 | -9,25 | -3,65 | 3,3 | 3,3 |
|  | CAD | 5 of 100 | -10,6 | -4,09 | 3,7 | 3,1 |
| hTAAR8_active-like_ | PUT | 6 of 83 | -9,26 | -3,81 | 3,6 | 3,7 |
|  | CAD | 1 of 100 | -10,12 | -4,28 | 3,3 | 3,4 |
| hTAAR8_inactive-like_ | PUT | 2 of 100 | -8,73 | -3,64 | 3,2 | 3,2 |
|  | CAD | 7 of 72 | -9,43 | -4,47 | 3,4 | 3,8 |
| zTAAR13c_active-like_ | CAD | 5 of 100 | -10,21 | -4,32 | 3,2 | 3,0 |
| zTAAR13c_inactive-like_ | CAD | 9 of 62 | -9,02 | -4,09 | 3,4 | 4,1 |
