# Supplementary material for: Identifying human diamine sensors for death related putrescine and cadaverine molecules
Source: PLoS Comput Biol. 2018 Jan 11;14(1):e1005945. doi: 10.1371/journal.pcbi.1005945 (PMC5783396; doi:10.1371/journal.pcbi.1005945)
Supplement: S3 Table — Ten molecular systems: hTAAR6active-like/PUT, hTAAR6active-like/CAD, hTAAR8active-like/PUT, hTAAR8active-like/CAD, zTAAR13cactive-like/CAD, hTAAR6inactive-like/PUT, hTAAR6inactive-like/CAD, hTAAR8inactive-like/PUT, hTAAR8inactive-like/CAD and zTAAR13cinactive-like/CAD were embedded in pre-equilibrated lipid bilayers containing 1-palmitoyl-2-oleoyl-sn-glycero-3-phosphatidylcholine (POPC), water molecules (TIP3P) and monoatomic Na+ and Cl- ions (0.2 M). All distances and RMSD values are shown in Angstroms (Å). *Reference experimental values DH-H POPC (303K): 37,0 [66]. (DOCX) [file pcbi.1005945.s012.docx]

| Conformational  State | MD  Systems | Box Size (x y z) | No of Lipids (POPC) | No of Waters | No of Ions (Na+/CL-) | Total Number of Atoms | DH-H (lipid bilayer thickness)* | RMSDbackbone (average) | RMSDligand (average) | Average distance  Asp_3.32_-Asp_5.42/5.43_ |
| --- | --- | --- | --- | --- | --- | --- | --- | --- | --- | --- |
| Active-like | TAAR6 / PUT | 88,6 88,6 150,4 | 169 | 27.112 | 176 / 182 | 96.764 | 45,0 | 3,1 | 1,7 | 8,9 |
|  | TAAR6 / CAD |  | 169 | 27.099 | 176 / 182 | 96.728 | 46,0 | 3,5 | 1,8 | 9,4 |
|  | TAAR8 / PUT |  | 171 | 27.078 | 177 / 182 | 96.744 | 46,4 | 2,8 | 2,0 | 9,2 |
|  | TAAR8 / CAD |  | 171 | 27.111 | 177 / 182 | 96.876 | 45,6 | 3,1 | 1,6 | 9,6 |
|  | TAAR13c / CAD |  | 167 | 27.120 | 177 / 182 | 96.754 | 43,7 | 2,5 | 1,5 | 9,8 |
|  |  |  |  |  |  |  |  |  |  |  |
| Inactive-like | TAAR6 / PUT | 82,3 78,5 112,3 | 180 | 14.752 | 69 / 76 | 58.390 | 40,0 | 3,1 | 2,9 | 10,4 |
|  | TAAR6 / CAD |  | 180 | 14.755 | 69 / 76 | 58.402 | 40,6 | 2,2 | 3,2 | 10,8 |
|  | TAAR8 / PUT |  | 178 | 14.780 | 70 / 75 | 58.369 | 40,3 | 2,6 | 2,6 | 11,2 |
|  | TAAR8 / CAD |  | 178 | 14.782 | 70 / 75 | 58.378 | 38,2 | 3,0 | 4,0 | 10,9 |
|  | TAAR13c / CAD |  | 171 | 14.654 | 70 / 75 | 58.209 | 39,6 | 2,7 | 2,0 | 10,7 |
